# Managing Supplier-Manufacturer Closed-Loop Supply Chain Considering Product Design and Take-Back Legislation

**DOI:** 10.3390/ijerph16040623

**Published:** 2019-02-20

**Authors:** Yue Wang, Baoying Xin, Zhe Wang, Bangyi Li

**Affiliations:** 1 College of Economics and Management, Nanjing University of Aeronautics and Astronautics, Nanjing 210016, China; wangyue0928@nuaa.edu.cn (Y.W.); libangyi@nuaa.edu.cn (B.L.); 2 Shandong Institute of New Urbanization, Shandong Management University, Jinan 250357, China; xinbaoying@sdmu.sdu.cn

**Keywords:** closed-loop supply chain, product design, take-back legislation

## Abstract

Facing a growing amount of waste electrical and electronic equipment (WEEE), a recent recast of the WEEE directive has put a specific reuse target for manufacturers, aiming to reduce environmental pollution and incentivize a green product design. In this paper, in order to examine whether the above two goals can be achieved by setting a specific reuse target, we have modelled a closed-loop supply chain consisting of a supplier (the leader) and a manufacturer (the follower) with the constraint of a mandated remanufacturing target. In this model, the supplier determines the level of interchangeability in product design and the wholesale price of the key component. The manufacturer buys the key components from the supplier and makes production and remanufacturing decisions under the requirement of a mandated remanufacturing target. We have investigated the supply chain’s members’ optimal decisions and analyzed the impact of the mandated remanufacturing target on the optimal profits of the supply chain’s members and consumer surplus, and finally, we have explored the environmental implications of the mandated remanufacturing target. We found that the supply chain’s members’ optimal decisions are affected by the mandated remanufacturing target and the cost of the new component. In terms of the economic implications of the mandated remanufacturing target, we have demonstrated that the increase in the mandated remanufacturing target has negative effects on the profits of the supply chain’s members and consumer surplus. Regarding the goal of incentivizing green product design, we found that the mandated remanufacturing target cannot always incentivize the supplier to implement product design that is beneficial to remanufacturing. From the perspective of the environment, we further indicate that more stringent mandated remanufacturing targets may bring an undesirable environmental outcome.

## 1. Introduction

In recent years, the rapid progress in technology brings a higher replacement frequency for electrical and electronic equipment (EEE), resulting in faster growth in the amount of waste electrical and electronic equipment (WEEE) each year. According to a forecast from the United Nations University, the world will accumulate 6.8 kg of e-waste per inhabitant by 2021 [1]. Traditional treatments of WEEE (including landfill and incineration) cause not only a waste of resource but also secondary pollution to the environment. In order to properly deal with the growing amount of WEEE, governments around the world have implemented various environmental legislation. Among them, product take-back legislation has been put forward based on the extended producer responsibility (EPR), requiring producers to hold physically or financially responsible for the proper treatment of end-of-life products [2]. Usually, product take-back legislation sets a minimum collection target for manufacturers. For example, the WEEE directive requires manufacturers to collect at least 45% of products sold by them. However, currently, a recent recast of the WEEE directive puts forward that a specific reuse target should be implemented to regulate the minimum quantity of used products that are required to be reused by the manufacturer [3]. Remanufacturing can restore the quality and function of used products to an as “new condition”, which is regarded as the most effective method for reuse. Thus, we set a mandated remanufacturing target in this paper to reflect the reuse target, which is consistent with the literature [3]. Although remanufacturing is considered as the most energy-efficient method, there is still doubt that setting remanufacturing targets is always beneficial to the environment. Thus, the first research objective of this paper is to explore the impact of setting a mandated remanufacturing target on the environment.

In fact, besides reducing harm to the environment, another objective of product take-back legislation is to incentivize the stakeholders to improve product design to make used products easier to be recovered [4]. Environmentally friendly product design can reduce recovery costs by incorporating ease for disassembly, reducing the use of hazardous materials and increasing the use of reusable materials [5]. Product design usually involves different design features, such as a design for interchangeability, a design for quality, and a design for modularity [6,7]. A design for interchangeability is a critical contributing factor in remanufacturing. It is difficult and costly to disassemble and remanufacture used products if these products lack an interchangeable design [6]. Thus, in this paper, we focus on the level of interchangeability in product design. The level of interchangeability in product design is defined as the degree of the product that can be disassembled, which is linked to the ease of remanufacturing. A higher level of interchangeability in product design indicates a greater salvageable value of the returned products because they are easier to be inspected, handled and disassembled.

Facing a mandated remanufacturing target, an interchangeable product design may benefit the manufacturer’s remanufacturing. However, an interchangeable product design would seem to hurt the supplier because of the cannibalization effect of the remanufactured components. Therefore, it is still unclear whether a mandated remanufacturing target can incentivize the supplier to implement an interchangeable design. Thus, the second research objective of this paper is to investigate the impact of an increased mandated remanufacturing target on the strategy of the supplier’s product design.

Multi-echelon supply chain management has garnered the focus of practitioners and academia. The implication of interactions across the supply chain is a key question in the multi-echelon supply chain. In this paper, we present a two-echelon supply chain consisting of a qualified EEE component supplier and a qualified EEE manufacturer. The supplier assumes the responsibility for product design for the EEE component and the manufacturer takes the responsibility for product recovery of its own WEEE. Considering the interactions between the supplier and the manufacturer, any decisions adopted by the supplier and manufacturer are dependent [8]. On the one hand, when the manufacturer engages in product recovery, the manufacturer can obtain the recovered components from WEEE, which can cannibalize market demands for the new components provided by the supplier [9]. On the other hand, the supplier can consequently react to the manufacturer’s product recovery decision by adjusting the wholesale price and the product design, which inversely influences the manufacturer’s decisions regarding new products and product recovery management. Thus, the third objective of this paper is to examine the impact of the interaction between the supplier and manufacturer in the context of take-back legislation.

In such a context, we seek to provide a better understanding on the following research questions: (1) In the context of product design and take-back legislation, what are the optimal decisions for the supplier and the manufacturer? (2) How do cost parameters affect the supplier’s and the manufacturer’s optimal decisions? (3) What are the impacts of the mandated remanufacturing target on the optimal decisions of supply chain partners and the consumer surplus? (4) What are the environmental implications and product design incentives for the mandated remanufacturing target?

To address the above research questions, we constructed a Stackelberg game model consisting of a qualified EEE component supplier and a qualified EEE manufacturer. The supplier is a leader who determines the level of interchangeability in product design and provides new key components to the manufacturer. The manufacturer is a follower who processes new products and sells them. As required by the take-back legislation on mandated remanufacturing targets, the manufacturer takes the initiative to remanufacture WEEE. The decision sequence between the manufacturer and the supplier is as follows: First, the supplier determines what kinds of product design of key components to implement and then sets the optimal level of interchangeability and the wholesale price of the key components. Then, based on the supplier’s optimal product design and wholesale price strategies, the manufacturer decides the optimal production strategies of new and remanufactured products.

By using Karush–Kuhn–Tucker (KKT) conditions to solve the game, we have characterized four optimal decisions of the manufacturer and the supplier and examined under what conditions they adopt different decisions. Then, we explored the impacts of the cost parameters on the optimal decisions using a sensitivity analysis. Subsequently, we discuss the impacts of the mandated remanufacturing target on optimal decisions of the supply chain partners and the consumer surplus. In particular, we stress on the effect of mandated remanufacturing targets on product design of the supplier to analyze the incentive mechanism of take-back legislation. To investigate the environmental implications of mandated remanufacturing targets, we have estimated the environmental impacts of each optimal decision and implemented a sensitivity analysis for the environmental impact of each decision.

The rest of this paper is organized as follows. In Section 2, we present an overview of relevant literature. Then, Section 3 introduces the modeling framework and formulates the model. In Section 4, we analyze the performances of the manufacturer and the supplier and implement a sensitivity analysis. In Section 5, we explore the impact of the mandated remanufacturing target on the supply chain’s decisions and consumer surplus. In Section 6, we investigate the environmental implications of the mandated remanufacturing target. Finally, Section 7 summarizes our findings and provides some managerial insights.

## 2. Literature Review 

Our study is mainly related to four streams of research literature. One falls into the stream of the applications of WEEE. The other falls into the stream of the interactions between supply chain partners on a closed loop supply chain. The third one falls into the stream of product design on remanufacturing. The fourth one falls into the stream of take-back legislation.

A great deal of efforts has been devoted to the applications of WEEE in order to properly deal with the WEEE. Many researchers have put forward that WEEE is valuable [10]. When WEEE has residual values, manufacturers and other stakeholders are interested in the recovery of WEEE. De Oliveira Neto et al. constructed the WEEE reverse logistics that consisted of three manufacturers and three recyclers to investigate their production and recycling decisions and assess their economic and environmental performances [11]. Based on the recycling of WEEE, Atasu and Subramanian investigated the impacts of collective and individual producer responsibility models of take-back laws on the manufacturer’s recycling decisions of WEEE and its economic performance [5]. The above literature considers the recycling of WEEE but does not take remanufacturing into account. In our paper, we regard remanufacturing as the only effective recovery method.

Many researchers have explored the interaction between supply chain partners on pricing, production, and recovery decisions of the closed loop supply chain. With regard to the interaction between the manufacturer and the retailer, Ma et al. constructed four reverse channel structures, namely, a central planner-collected, a manufacturer-collected, a retailer-collected and a third party-collected, and then investigated the optimal marketing effort, collection rate and pricing decisions of all players in the supply chain [12]. This paper assumes that the manufacturer is a leader when taking the interactions between the manufacturer and the retailer into account. In contrast to the above literature, the following research literature assumes that the retailer is a leader when considering the interactions between the manufacturer and the retailer. Sadjadi et al. considered a model consisting of two manufacturers and a retailer who is the leader and have investigated the impacts of competition between them on the pricing and service decisions of the supply chain [13]. Giri and Maiti studied a retailer-led Stackelberg game in a multi-echelon supply chain involving a supplier, a manufacturer and a retailer and explored how to improve the profits of the supply chain through the game [14]. This paper considers the interactions between the supplier and the manufacturer. Kim and Ouardigh also considered a collaboration between a manufacturer and a supplier and investigated how to allocate resources between improving existing products and developing new products [15]. This paper focuses on the impact of the interactions between the manufacturer and the supplier on production decisions without considering product recovery management. Zhou et al. considered a decentralized closed-loop supply chain consisting of a manufacturer and a supplier and then analyzed the impacts of internal conflict on the players’ optimal decisions in regards to remanufacturing [16]. Xiong et al. considered two forms of remanufacturing (manufacturer-remanufacturing and supplier-remanufacturing), and then analyzed the performance of these players in a decentralized closed-loop supply chain [17]. These papers take remanufacturing into account and investigate the impact of the interaction on the remanufacturing decisions. In our paper, we construct a two-echelon supply chain model consisting of a manufacturer and a supplier who is a leader and comprehensively take collection, remanufacturing and disposal into account.

A large number of studies have concentrated on the product design in a closed loop supply chain. Shi et al. investigated the effect of a remanufacturable product design on the market segmentation and pricing decisions of the manufacturer [18]. They only regard the manufacturer as the subject and ignore the other partners in the closed loop supply chain. Wu introduced competition between a remanufacturer and a manufacturer and examined the production decision of the manufacturer and the competitive pricing strategy [6]. The impacts of the interaction between the retailer and the manufacturer on the product design have also been highlighted by several studies. Hua et al. modelled a two-echelon supply chain consisting of a manufacturer and a retailer and investigated the optimal green product design strategy of the manufacturer [19]. Based on the manufacturer-retailer supply chain, Zhu and He investigated the impacts of supply chain structures, green product types and competition types on the green product design decisions [20]. The above literature does not consider the interactions between the supplier and the manufacturer. In this paper, we assume that the supplier is responsible for product design and investigate the optimal supplier’s and manufacturer’s decisions in the context of the interactions between the supplier and the manufacturer. 

To our knowledge, there are several studies focusing on the impacts of take-back legislation. By setting collection and recycling targets, Huang et al. explored the impact of take back legislation based on extended producer responsibility on the product design of the manufacturer [21]. Jacobs and Subramanian constructed a two-echelon model consisting of a supplier and a manufacturer and analyzed the impact of take back legislation on recycling decisions in the integrated and decentralized supply chain [8]. These two papers both take collection and recycling targets into account but do not consider a reuse target. Based on the existing forms of e-waste legislations, Shumail et al. proposed a new form of e-waste legislation with an additional reuse target and compared their economic and environmental performance [22]. Esenduran et al. specified a reuse target as a remanufacturing target and studied the monopoly manufacturer’s decision in response to take back legislation as well as the environmental implications of take back legislation [3]. Then, Esenduran et al. enriched their study by incorporating competition between the manufacturer and the remanufacturer [23]. In this paper, we have also studied the economic and environmental implications of take back legislation with the mandated remanufacturing target.

The differences between our paper and the existing literature are as follows. (1) We constructed a two-echelon supply chain consisting of a supplier and a manufacturer and studied the impacts of the interactions between them on remanufacturing. (2) We assumed that the product design of the supplier is an endogenous variable and explored the optimal product design in the context of the interaction between the supplier and the manufacturer and take-back legislation. (3) We incorporated the mandated remanufacturing target and investigated the economic and environmental implications of the mandated remanufacturing target in a supplier-manufacturer supply chain. 

## 3. Modeling Framework

In this section, we identify the decision-making framework and introduce key assumptions and notations concerning consumer preferences and the cost structure. The main parameters and notations are summarized in Table 1.

We assume that the market size is Q and each consumer buys at most one unit in a single period. Consumers are heterogeneous and their willingness to pay v is uniformly distributed in the interval (0,Q). Consistent with the literature [24,25], consumers do not view remanufactured products as perfect substitutes for new products; although remanufactured products can display similar performances as new products can. Thus, if the consumer’s valuation for a new product is denoted by ν, then the consumer’s valuation for the remanufactured product is denoted by αν, where α represents the consumer value discount for the remanufactured product. Let $p_{n}$ and $q_{n}$, and $p_{r}$ and $q_{r}$ denote the price and demand for new and remanufactured products, respectively. If the consumer buys a new product, he can get utility $U_{n}=\nu -p_{n}$ from the new product. If the consumer buys a remanufactured product, he can get utility $U_{r}=\alpha \nu -p_{r}$ from the remanufactured product. When $U_{n}>0$ and $U_{n}\geq U_{r}$, the consumers purchase new products. When $U_{r}>0$ and $U_{r}>U_{n}$, the consumers purchase remanufactured products. To ensure that there are remanufactured products on the market, the remanufactured product adopts a low price strategy, that is $p_{r}<\alpha p_{n}$ [26,27,28]. According to the utility functions, the inverse demand functions for new and remanufactured products are $p_{n}=Q-q_{n}-\alpha q_{r}$ and $p_{r}=\alpha \left(Q-q_{n}-q_{r}\right)$, respectively [29,30].

We have constructed a two-echelon closed supply chain model consisting of one qualified EEE manufacturer and one qualified EEE component supplier in a single period. In a steady single period, the supplier holds the responsibility for product design of the key components besides selling key components to the manufacturer. After obtaining new components from the supplier, the manufacturer assembles, tests and makes them ready for sale as new products. In terms of WEEE (we use “used products” or “collected cores” to represent WEEE in the following paper), the manufacturer collects the used products to remanufacture key components and then uses the remanufactured components to produce remanufactured products. Consistent with the literature [3,23], in order to improve the utilization of the resources, the government, as a regulator, requires the manufacturer to meet the lowest remanufacturing target, γ. The manufacturer must remanufacture at least a fraction γ of used products to fulfill the government’s environmental goal. In addition, referring to the literature [7,31,32], in a single period, new products sold earlier are available for remanufacturing, so the quantity of remanufactured product is bound by the quantity of new product sold, namely $q_{r}\leq q_{n}$. 

We considered that one new (remanufactured) product only needs one new (remanufactured) key component. Let the unit cost and the wholesale price of the new component be denoted by $c_{n}$ and w, respectively. Let the unit cost of the remanufactured component be denoted by $c_{r}$. Besides the cost to obtain the new component w and the cost of the remanufactured component $c_{r}$, we normalized the other costs of assembly, testing and processing to 0. 

We assumed that the product design of the supplier can be reflected by the level of interchangeability in the new key component [19]. The level of interchangeability in the new component is represented by ω and is determined by the ease of remanufacturing. If ω=0, the supplier adopts no product design strategy. If ω>0, the supplier adopts a product design strategy that is beneficial to assemble and remanufacture the product by increasing the disassembly efficiency and reducing the costs of remanufacturing. If ω<0, the supplier implements a product design strategy that is not beneficial to assemble and remanufacture the product. The higher ω, the more beneficial for the manufacturer to remanufacture. The lower ω, the harder for the manufacturer to remanufacture. The cost of key component’s product design is denoted by the nonlinear function $\frac{1}{2}\kappa \omega^{2}$. This demonstrates that the cost of product design is a convex-increasing function of the level of interchangeability ω, indicating that a higher positive level of interchangeability or lower negative level of interchangeability needs more effort [6,33,34,35]. The supplier’s responsibility for product design has a significant impact on the ease of the remanufacturing of used products, which can be represented by the impact of the level of interchangeability in the new component on the cost of remanufacturing. If the level of interchangeability in the new component is positive and high, the components of the used products are easier to be remanufactured, thereby decreasing the cost of remanufactured the product. We assumed that if the supplier increases one unit of the level of interchangeability in the key component, then the manufacturer can save Δ unit of remanufacturing costs. Δ represents the saving of remanufacturing costs due to product design. Therefore, Δ>0. Thus, considering the supplier’s product design of the new component, the unit cost of the remanufactured component can be denoted by $c_{r}-\Delta \omega$.

Thus, the supplier’s profit function is presented as follows:

(1) $$\Pi_{S}^{}\left(w,\omega \right)=\left(w-c_{n}\right)q_{n}-\frac{1}{2}\kappa \omega^{2}.$$

The manufacturer’s profit function is presented as follows:

(2) $$\Pi_{M}^{}\left(q_{n},q_{r}\right)=q_{n}\left(p_{n}-w\right)+q_{r}\left(p_{r}-c_{r}+\Delta \omega \right)$$

(3) $$s.t.\;\;\;\;\gamma q_{n}\leq q_{r}\leq q_{n}.$$

We constructed a Stackelberg game model consisting of a supplier and a manufacturer. The supplier is a leader and the manufacturer is a follower. The decision sequence is as follows: First, the supplier determines whether or not to implement the product design for the key components, and then sets the optimal level of interchangeability and the wholesale price of the key components. Then, based on the supplier’s optimal product design and wholesale price strategies, the manufacturer decides the optimal production strategies for new and remanufactured products. By using backward induction, we first solved the manufacturer’s optimal equilibrium quantities of new and remanufactured products under take-back legislation. Then, given certain optimal strategies of the manufacturer, we can further determine the supplier’s optimal level of interchangeability and the wholesale price of key components.

## 4. Model Analysis

In this section, we investigated the supplier’s wholesale pricing and product design strategies and the manufacturer’s production strategies in a two-echelon closed loop supply chain. First, based on the principles of profit maximization, we determined the optimal equilibrium solutions of the supplier and the manufacturer. Then, we further explored the impact of main cost parameters on the product design, production decisions and profits.

### 4.1. Characterization of Optimal Equilibrium

By using backward induction, we first decided the optimal production quantities of the new product and remanufactured product to maximize the manufacturer’s profit given a certain w and ω.

Proposition 1. Given the supplier’s optimal wholesale price and product design for the key component, the manufacturer’s optimal production quantities of new products and remanufactured products are:

(i) Decision MA,

$q_{n}^{MA}=\frac{Q-w+\left(Q\alpha +\Delta \omega -c_{r}\right)\gamma }{2+2\alpha \gamma \left(2+\gamma \right)}$, $q_{r}^{MA}=\frac{\gamma \left(Q-w+\left(Q\alpha +\Delta \omega -c_{r}\right)\gamma \right)}{2+2\alpha \gamma \left(2+\gamma \right)}$,

when $c_{n}<w<\frac{c_{r}-\Delta \omega +\alpha \left(Q-Q\alpha -\Delta \omega +c_{r}\right)\gamma }{\alpha \left(1+\gamma \right)}$;

(ii) Decision MB,

$q_{n}^{MB}=\frac{Q\left(1-\alpha \right)-w+c_{r}-\Delta \omega }{2\left(1-\alpha \right)}$, $q_{r}^{MB}=\frac{w\alpha +\Delta \omega -c_{r}}{2\left(1-\alpha \right)\alpha }$,

when $\frac{c_{r}-\Delta \omega +\alpha \left(Q-Q\alpha -\Delta \omega +c_{r}\right)\gamma }{\alpha \left(1+\gamma \right)}\leq w\leq \frac{Q\left(1-\alpha \right)\alpha +\left(1+\alpha \right)(c_{r}-\Delta \omega )}{2\alpha }$;

(iii) Decision MC,

$q_{n}^{MC}=\frac{Q-w+Q\alpha -c_{r}+\Delta \omega }{2\left(1+3\alpha \right)}$, $q_{r}^{MC}=\frac{Q-w+Q\alpha -c_{r}+\Delta \omega }{2\left(1+3\alpha \right)}$,

when $\frac{Q\left(1-\alpha \right)\alpha +\left(1+\alpha \right)(c_{r}-\Delta \omega )}{2\alpha }<w\leq Q+Q\alpha +\Delta \omega -c_{r}$.

For proof, see Appendix A.1.

When given a level of interchangeability in the product design and the wholesale price of key components, the supplier needs to refer to the manufacturer’s optimal production decisions in proposition 1. If the supplier expects that the manufacturer will adopt decision MA, then the corresponding decision of the supplier is represented as decision SA. Then, the corresponding profit function of the supplier is $\Pi_{S}^{}\left(w^{SA},\;\omega^{SA}\right)=\left(w^{SA}-c_{n}\right)q_{n}^{MA}-\frac{1}{2}\kappa {\left(\omega^{SA}\right)}^{2}$, which subjects to $c_{n}<w^{MA}<\frac{c_{r}-\Delta \omega +\alpha \left(Q-Q\alpha -\Delta \omega +c_{r}\right)\gamma }{\alpha \left(1+\gamma \right)}$. This constraint ensures that the manufacturer adopts decision MA. Considering all possible decisions that can be adopted by the manufacturer, we can correspondingly obtain the supplier’s optimal wholesale pricing and product design.

Proposition 2. For the given the manufacturer’s optimal decisions in proposition 1, the supplier’s optimal decisions are as follows:

(i) If $0<\gamma <\gamma^{*}$,

when $c_{r}<c_{n}\leq A$, the supplier chooses decision SA;

when $A<c_{n}\leq B_{1}$, the supplier chooses decision SB-1;

when $B_{1}<c_{n}\leq K_{1}$, the supplier chooses decision SB-2;

when $K_{1}<c_{n}\leq C_{2}$, the supplier chooses decision SC.

(ii) If $\gamma^{*}\leq \gamma <1$, 

when $c_{r}<c_{n}\leq A$, the supplier chooses decision SA;

when $A<c_{n}\leq K_{2}$, the supplier chooses decision SB-1;

when $K_{2}<c_{n}\leq C_{2}$, the supplier chooses decision SC;

The optimal wholesale price and product design under the above supplier’s decisions are shown in Table 2. Here, $\gamma^{*}=\frac{\sqrt{\alpha^{2}\left(4\left(1-\alpha \right)\kappa -\Delta^{2}\right)\left(4\left(\kappa +3\alpha \kappa \right)-\Delta^{2}\right)}-\alpha \left(\Delta^{2}+4\kappa \right)}{4\alpha^{2}\kappa }$, $H_{1}=Q-c_{n}$ and $H_{2}=Q\alpha -c_{r}$.

For proof, see Appendix A.2.

Proposition 2 shows that the mandated remanufacturing target affects the supplier’s optimal decisions. If the mandated remanufacturing target is low, namely $0<\gamma <\gamma^{*}$, the supplier has four optimal pricing and product design decisions based on the cost of the new component. If the mandated remanufacturing target is high, namely $\gamma^{*}\leq \gamma <1$, the supplier has three optimal pricing and product design decisions. In contrast to the conditions under the low mandated remanufacturing target, the supplier gives up decision SB-2 under a high mandated remanufacturing target, indicating that the manufacturer does not remanufacture part of the used products voluntarily. This is because the high mandated remanufacturing target leads to no chance for the manufacturer to remanufacture voluntarily.

When the cost of a new component is low enough ($c_{r}<c_{n}\leq A$), the supplier’s optimal decision is SA. Under decision SA, the supplier’s level of interchangeability in the key component $\omega^{SA}$ is positive, which means that the supplier’s product design is beneficial for remanufacturing. In this situation, the manufacturer only remanufactures the mandated quantity of used products in order to meet the mandated remanufacturing target. 

When the cost of the new component is low ($A<c_{n}\leq \min\{B_{1},K_{2}\}$), the supplier’s optimal decision is SB-1. Under this decision, the supplier’s level of interchangeability in the new component $\omega^{SB-1}$ is closely related to the cost of the new component. When the cost of the new component is lower than $c_{n1}$, the product design that the supplier implements is beneficial for remanufacturing. When the cost of the new component is higher than $c_{n1}$, the product design that the supplier implements is harmful to remanufacturing. Here, $c_{n1}=\frac{\alpha \left(Q-2Q\alpha +2c_{r}\right)\gamma -Q\alpha +2c_{r}}{\alpha \left(1+\gamma \right)}$. In this situation, the manufacturer still only remanufactures the mandated quantity of used products just in order to meet the government’s requirements. 

When the cost of the new component is moderate ($B_{1}<c_{n}\leq K_{1}$), the supplier’s optimal decision is SB-2. Under this decision, the supplier’s level of interchangeability in the new component $\omega^{SB-2}$ is negative, which means that the supplier’s product design is harmful to remanufacturing. In this situation, the manufacturer is willing to remanufacture more used products. The voluntary remanufacturing rate exceeds the mandated remanufacturing target, so the mandated remanufacturing target is ineffective in this situation.

When the cost of the new component is high enough ($\max\{K_{1},\;K_{2}\}<c_{n}\leq C_{2}$), the supplier’s optimal decision is SC. At this time, the supplier’s level of interchangeability in the key component $\omega^{SC}$ is positive, which means that the supplier’s product design is beneficial to remanufacturing. In this situation, the manufacturer remanufactures all used products. The voluntary remanufacturing rate is equal to 1. 

It is noteworthy that when $Q\leq c_{n}\leq C_{2}$, producing new products alone cannot obtain profits but it can provide enough used products for remanufacturing. Remanufacturing is still profitable and the profits due to the sale of remanufactured products can compensate for the loss due to sales of new products. When $c_{n}>C_{2}$, remanufacturing is not profitable; thus the production of new products ceases.

Substitute the optimal wholesale price and product design into the manufacturer’s optimal decisions in proposition 1. The manufacturer’s optimal production decisions are shown in Table 3.

### 4.2. Sensitivity Analysis

In this section, we mainly investigated the impacts of changes in the cost parameters on the supplier’s and the manufacturer’s optimal decisions. For the convenience of the analysis, we used the subscript DA, DB-1, DB-2 and DC to represent the supplier’s and the manufacturer’s common optimal decisions.

#### 4.2.1. The Impacts of Cost Parameters on the Supplier’s Product Design and Wholesale Price

Proposition 3. (i) A sensitivity analysis of optimal product design under different decisions is shown in Table 4, where the signs +, −, 0 indicate an increase, decrease and no change in optimal solutions respectively. 

(ii) If $0<\gamma <\gamma^{*}$, $\omega^{DA}\left(A\right)=\omega^{DB-1}\left(A\right)$, $\omega^{DB-1}\left(B_{1}\right)=\omega^{DB-2}\left(B_{1}\right)$, $\omega^{DB-2}\left(K_{1}\right)<\omega^{DC}\left(K_{1}\right)$. If $\gamma^{*}\leq \gamma <1$, $\omega^{DA}\left(A\right)=\omega^{DB-1}\left(A\right)$, $\omega^{DB-1}\left(K_{2}\right)<\omega^{DC}\left(K_{2}\right)$.

For proof, see Appendix A.3.

When the cost of the new component is low enough, namely Ddecision DA, with the constraint of the mandated remanufacturing target, the quantity of remanufactured product depends on the quantity of new products. The manufacturer is passively required to remanufacture. Meanwhile, the complementary between the remanufactured products and the new products is prominent. At this time, the product design that the supplier implements is beneficial to remanufacturing, that is $\omega^{DA}>0$. Therefore, along with the increase in $c_{n}$ and $c_{r}$, the supplier decreases the level of interchangeability in the new component to reduce the investment cost of the product design and further the loss of profit. Otherwise, the supplier improves the product design to increase the level of interchangeability in the new component.

When the cost of the new component is low, namely decision DB-1, with the constraint of the mandated remanufacturing target, the quantity of the remanufactured product depends on the quantity of new products. The manufacturer is still passively required to remanufacture. Although the remanufacturing cost advantage improves with the increased cost of the new component, the complementary between remanufactured products and new products is still prominent. Both the decrease in the profit of new products and the increase in the profit of remanufactured products lead to the supplier decreasing the level of interchangeability in the new component in case of decreased demand for new products. Noteworthy, the supplier’s level of interchangeability in the new component changes from positive to negative as the cost of new component increases. When $c_{n}=c_{n1}$, $\omega^{DB-1}(c_{n1})=0$, which demonstrates that the supplier gives up any kind of product design. In addition, when $c_{n}<c_{n1}$, the level of interchangeability ($\omega^{DB-1}>0$) is a concave increasing function of Δ. This means that when the remanufacturing cost advantage is negligible, with an increase in Δ, the supplier improves the level of interchangeability to increase the manufacturer’s demand for new products. When $c_{n}>c_{n1}$, the level of interchangeability ($\omega^{DB-1}<0$) is a convex decreasing function of Δ. This indicates that as the remanufacturing cost advantage improves, the increase in Δ spurs the supplier to implement product design that is unfavorable for remanufacturing to deter the manufacturer from remanufacturing.

When the cost of a new component is moderate, namely decision DB-2, the manufacturer voluntarily collects part of the used products for remanufacturing. The competitiveness between new products and remanufactured products is dominant. In order to prevent the manufacturer’s remanufacturing, the product design that the supplier implements is harmful to remanufacturing; that is $\omega^{DB-2}<0$. However, as the cost of new component increases, the profit of new products decreases, weakening the deterrent effects of the supplier’s product design on the manufacturer’s remanufacturing. Thus, in order to reduce one’s own profit loss from the cost of product design that is harmful to remanufacturing, the supplier enhances the negative level of interchangeability in the new component. The higher the cost of remanufacturing, the less profit there is in the remanufactured products. Then, the supplier decreases the negative level of interchangeability, thereby preventing the manufacturer from remanufacturing.

When the cost of the new component is high, namely decision DC, the remanufacturing cost advantage is very prominent. Therefore, the manufacturer is willing to remanufacture all used products. The quantity of remanufactured products depends on the quantity of new products, thus the complementary between the remanufactured products and the new products is dominant. At this time, a product design that the supplier implements is beneficial for remanufacturing, namely $\omega^{DC}>0$. Therefore, decreases in the profits of remanufactured and new products both can lead to a supplier decreasing the level of interchangeability in the new component to reduce the loss of profit. Otherwise, the supplier improves the level of interchangeability in the new component to increase profits.

**Figure 1:**
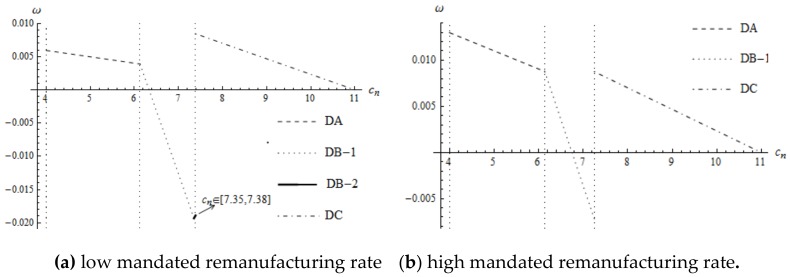
The impact of $c_{n}$ on the supplier’s product design decision.

According to Proposition 3(ii) and Figure 1, when the supplier’s optimal decision is decision DC, a product design that the supplier implements is beneficial to remanufacturing. However, whether decision DB-2 under low remanufacturing target or decision DB-1 under a high remanufacturing target, the product design that the suppliers both implement is harmful to remanufacturing. Once the optimal decision changes from decision DB to decision DC, the level of interchangeability in the new component changes from positive to negative rapidly. Therefore, $\omega^{DB-2}\left(K_{1}\right)<\omega^{DC}\left(K_{1}\right)$ and $\omega^{DB-1}\left(K_{2}\right)<\omega^{DC}\left(K_{2}\right)$.

Proposition 4. (i) Sensitivity analysis of optimal wholesale price under different decisions is shown in the Table 5, where the signs +, −, 0 indicate an increase, decrease and no-change in equilibrium, respectively.

(ii) If $0<\gamma <\gamma^{*}$, $w^{DA}\left(A\right)=w^{DB-1}\left(A\right)$, $w^{DB-1}\left(B_{1}\right)=w^{DB-2}\left(B_{1}\right)$, $w^{DB-2}\left(K_{1}\right)<w^{DC}\left(K_{1}\right)$. If $\gamma^{*}\leq \gamma <1$, $w^{DA}\left(A\right)=w^{DB-1}\left(A\right)$, $w^{DB-1}\left(K_{2}\right)<w^{DC}\left(K_{2}\right)$.

For proof, see Appendix A.4.

The wholesale price is increasing in the cost of the new component ($c_{n}$). This is consistent with our intuition that the higher cost of the new component is, the higher the wholesale price is.

**Figure 2:**
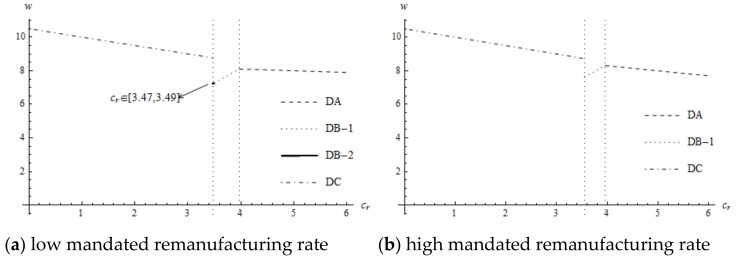
The impact of $c_{r}$ on the supplier’s wholesale price.

The impact of the cost of remanufacturing ($c_{r}$) on the supplier’s wholesale price is more complicated, as is shown in Figure 2. When the cost of the new component is low enough (decision DA), the complementary between new and remanufactured products is prominent. As the cost of remanufacturing increases, the degree of difficulty for remanufacturing increases, resulting in decreased demands for new components. Thus, the supplier decreases the wholesale price to expand the sale of new components to gain more profit. When the cost of the new component is low (decision DB-1), although the demands for new products depend on the mandated remanufacturing target, the manufacturer’s willingness to remanufacture enhances. The supplier with an increased component cost improves the wholesale price to capture more profit as $c_{r}$ increases. When the cost of new components is moderate (decision DB-2), with an increase in $c_{r}$, the profit of remanufacturing becomes less, weakening the competitiveness between new and remanufactured products. Therefore, the supplier with an increased component cost improves the wholesale price to obtain more profit. When the cost of new components is high enough (decision DC), the manufacturer voluntarily remanufactures all used products. The complementary between remanufactured and new products dominates. With an increase in $c_{r}$, in order to sell more new components, the supplier decreases the wholesale price of the new components to incentivize the manufacturer to remanufacture.

The monotonicity of the supplier’s wholesale price regarding Δ is affected by the level of interchangeability in the new component. Under decision DA, the increase in Δ means saving more remanufacturing costs due to product design, thus the supplier increases the wholesale price to deter the manufacturer from remanufacturing. Under decision DB-1, $c_{n1}$ is a trade-off point of the level of interchangeability. When $c_{n}<c_{n1}$, the level of interchangeability in product design is positive, indicating that the product design is beneficial to remanufacturing, thus the supplier increases the wholesale price. When $c_{n}>c_{n1}$, the level of interchangeability in product design is negative, indicating that the product design is harmful to remanufacturing, thus the supplier decreases the wholesale price. Both under decision DB-2 and decision DC, the cost of new components is so high that the remanufacturing cost advantage is prominent, thus the supplier increases the wholesale price to keep the manufacturer from remanufacturing.

Noteworthy, Proposition 4 (ii) indicates that, once the supplier’s optimal decision changes from decision DB-1 or decision DB-2 to decision DC, the wholesale price increases rapidly. This is because that mandated remanufacturing target of the strengthened competitiveness between new and remanufactured products limits the increase in the wholesale price. Once the optimal decision is DC, the complementary between new and remanufactured products dominates, resulting in a substantial increase in the wholesale price to obtain more profit. Hence, $K_{1}$ and $K_{2}$ are piecewise points of the supplier’s wholesale price.

#### 4.2.2. The Impacts of Cost Parameters on the Manufacturer’s Production Decisions 

Proposition 5. (i) A sensitivity analysis of the optimal quantity of new product and remanufactured product under different strategies is shown in the Table 6, where the signs +, −, 0 indicate an increase, decrease and no-change in equilibrium, respectively.

(ii) If $0<\gamma <\gamma^{*}$, $q_{n}^{DA}\left(A\right)=q_{n}^{DB-1}\left(A\right)$, $q_{n}^{DB-1}\left(B_{1}\right)=q_{n}^{DB-2}\left(B_{1}\right)$, $q_{n}^{DB-2}\left(K_{1}\right)>q_{n}^{DC}\left(K_{1}\right)$;$q_{r}^{DA}\left(A\right)=q_{r}^{DB-1}\left(A\right)$, $q_{r}^{DB-1}\left(B_{1}\right)=q_{r}^{DB-2}\left(B_{1}\right)$, $q_{r}^{DB-2}\left(K_{1}\right)<q_{r}^{DC}\left(K_{1}\right)$. If $\gamma^{*}\leq \gamma <1$, $q_{n}^{DA}\left(A\right)=q_{n}^{DB-1}\left(A\right)$, $q_{n}^{DB-1}\left(K_{2}\right)>q_{n}^{DC}\left(K_{2}\right)$; $q_{r}^{DA}\left(A\right)=q_{r}^{DB-1}\left(A\right)$, The relationship between $q_{r}^{DB-1}\left(K_{2}\right)$ and $q_{r}^{DC}\left(K_{2}\right)$ is uncertain.

For proof, see Appendix A.5.

In terms of $c_{n}$ and $c_{r}$, under decision DA, DB-1 and DC, the quantity of remanufactured products depends on the quantity of new products, thus the complementary between them is relatively prominent. Therefore, both the quantities of new and remanufactured products decrease with the increase in $c_{n}$ and $c_{r}$. Under decision DB-2, the competitiveness between new and remanufactured products is relatively prominent. Therefore, the quantity of new product is increasing in $c_{r}$, whereas the quantity of remanufactured products is increasing in $c_{n}$.

In terms of Δ, under decision DA and DC, the quantity of remanufactured product depends on the quantity of new product and they are complements. Thus, the quantities of both new and remanufactured products are increasing in Δ. Under decision DB-1, the monotonicities of the quantities of the new and remanufactured products regarding Δ are affected by the supplier’s product design. When $c_{n}<c_{n1}$, the level of interchangeability in product design is positive, and the quantities of new and remanufactured products are increasing in Δ. When $c_{n}>c_{n1}$, the level of interchangeability in product design is negative, and the quantities of new and remanufactured products are decreasing in Δ. Under decision DB-2, in order to prevent the manufacturer from remanufacturing, the supplier implements a product design that is harmful to remanufacturing, that is, the level of interchangeability in product design is negative. Thus, the quantity of new product is increasing in Δ whereas the quantity of remanufactured product is decreasing in Δ.

**Figure 3:**
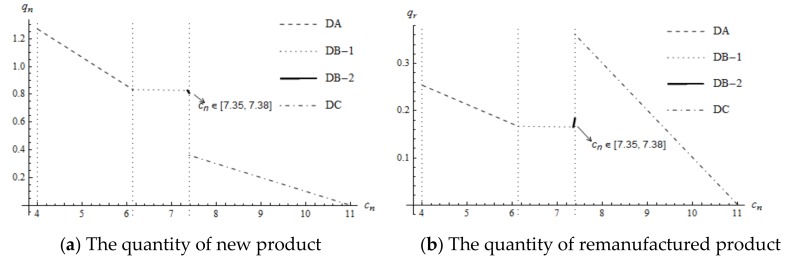
The impact of $c_{n}$ on the manufacturer’s production decision under low mandated remanufacturing target.

Based on Proposition 4, when the supplier’s optimal decision changes from DB-2 to DC, the wholesale price increases quickly, leading to decreased demands for new components. Thus, the quantity of new products decreases rapidly whereas the quantity of remanufactured products increases rapidly. As shown in Figure 3, $K_{1}$ is a piecewise point.

**Figure 4:**
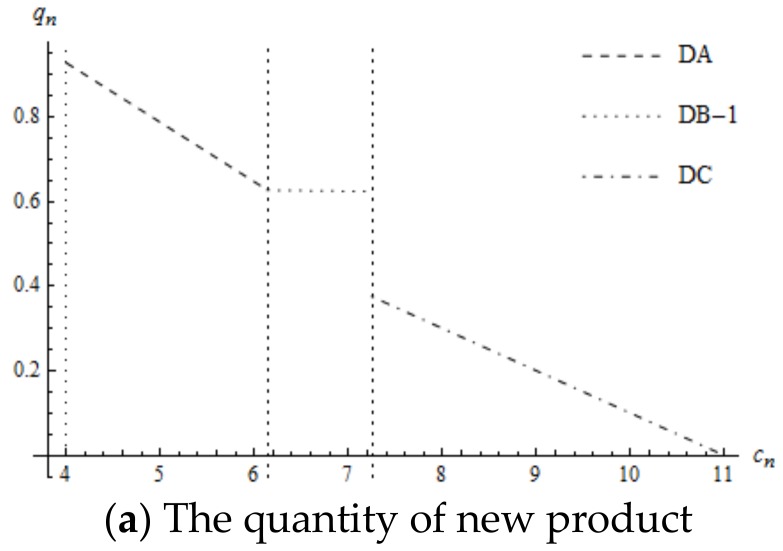

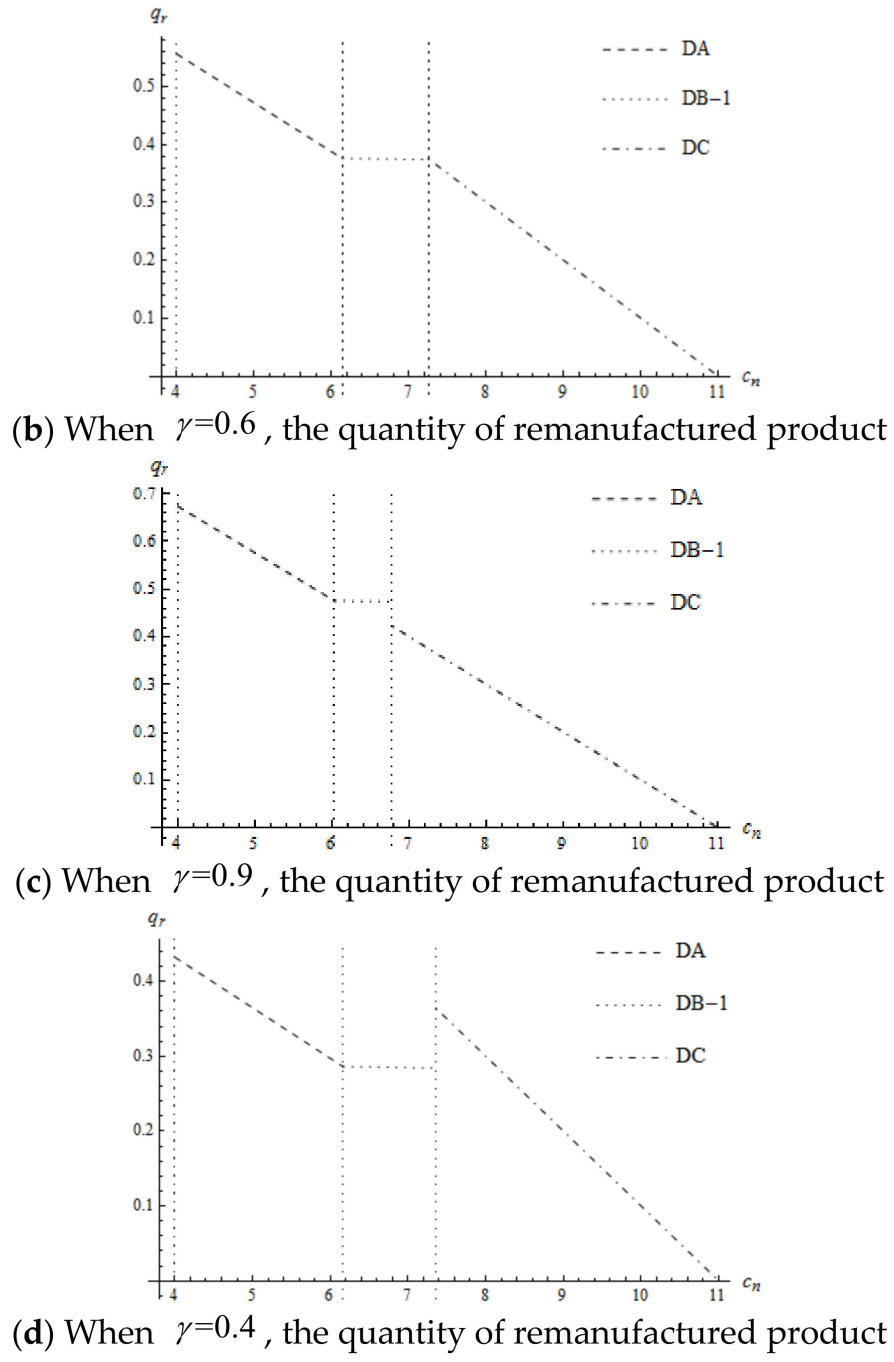
The impact of $c_{n}$ on the manufacturer’s production decision under high mandated remanufacturing target.

Under a high mandated remanufacturing target, once the manufacturer’s optimal decisions switch from DB-2 to DC, the quantity of new products reduces quickly but the change in the quantity of the remanufactured product is affected by the remanufacturing target. As shown in Figure 4.

Proposition 6. (i) A sensitivity analysis of the supplier’s and the manufacturer’s optimal profits under different strategies is shown in the Table 7, where the signs +, −, 0 indicate an increase, decrease and no-change in equilibrium, respectively.

(ii) If $0<\gamma <\gamma^{*}$, $\Pi_{S}^{DA}\left(A\right)=\Pi_{S}^{DB-1}\left(A\right)$, $\Pi_{S}^{DB-1}\left(B_{1}\right)=\Pi_{S}^{DB-2}\left(B_{1}\right)$, $\Pi_{S}^{DB-2}\left(K_{1}\right)=\Pi_{S}^{DC}\left(K_{1}\right)$;$\Pi_{M}^{DA}\left(A\right)=\Pi_{M}^{DB-1}\left(A\right)$, $\Pi_{M}^{DB-1}\left(B_{1}\right)=\Pi_{M}^{DB-2}\left(B_{1}\right)$, $\Pi_{M}^{DB-2}\left(K_{1}\right)>\Pi_{M}^{DC}\left(K_{1}\right)$. If $\gamma^{*}\leq \gamma <1$, $\Pi_{S}^{DA}\left(A\right)=\Pi_{S}^{DB-1}\left(A\right)$, $\Pi_{S}^{DB-1}\left(K_{2}\right)=\Pi_{S}^{DC}\left(K_{2}\right)$; $\Pi_{M}^{DA}\left(A\right)=\Pi_{M}^{DB-1}\left(A\right)$, $\Pi_{M}^{DB-1}\left(K_{2}\right)>\Pi_{M}^{DC}\left(K_{2}\right)$.

For proof, see Appendix A.6.

Firstly, we analyze the impact of the cost parameters on the supplier’s profit. In any case, the supplier’s profit is always decreasing in $c_{n}$ because the supplier’s profit originates only from the sale of new components. In addition, the supplier’s profit is affected by $c_{r}$. Under decision DA and decision DC, the complementary between new and remanufactured products is prominent, and the increase in $c_{r}$ leads to decreased demands for the new components, thereby the supplier’s profit is decreasing. Under decision DB-1, although the quantities of new and remanufactured products decrease with an increase in $c_{r}$, the supplier’s profit is influenced by the level of interchangeability in product design. When $c_{n}<c_{n1}$, the level of interchangeability in product design is positive, strengthening the competitiveness of the remanufactured products. Thus, the supplier’s profit is decreasing in $c_{r}$. When $c_{n}>c_{n1}$, the level of interchangeability in product design is negative, weakening the competitiveness of the remanufactured products. Thus, the supplier’s profit is increasing in $c_{r}$. Under decision DB-2, new products and remanufactured products strongly compete with each other. The level of interchangeability in product design is negative. As $c_{r}$ increases, the supplier’s profit increases. Under all decisions, all the supplier’s profits are increasing in Δ.

Secondly, we analyzed the impact of the cost parameters on the manufacturer’s profit. The manufacturer’s profit is decreasing not only in $c_{r}$ but also in $c_{n}$. This is because the manufacturer’s profits come both from the sale of new products and the sale of remanufactured products. The remanufacturing saving cost due to the product design (Δ) has an impact on the manufacturer’s profit. Under decision DA and decision DC, the complementary between new and remanufactured products is prominent. Therefore, an increase in Δ leads to increased demands for new products and remanufactured products, thereby the manufacturer’s profit increasing. Under decision DB-1, the manufacturer’s profit is influenced by the supplier’s product design decision. When $c_{n}<c_{n1}$, the level of interchangeability in product design is positive, improving the demands for new and remanufactured products. Thus, the supplier’s profit is increasing in Δ. When $c_{n}>c_{n1}$, the level of interchangeability in product design is negative, decreasing the demands for new and remanufactured products. Thus, the supplier’s profit is decreasing in Δ. Under decision DB-2, new products and remanufactured products strongly compete with each other. The level of interchangeability in product design is negative. It is hard for the manufacturer to remanufacture, thus his profit decreases.

**Figure 5:**
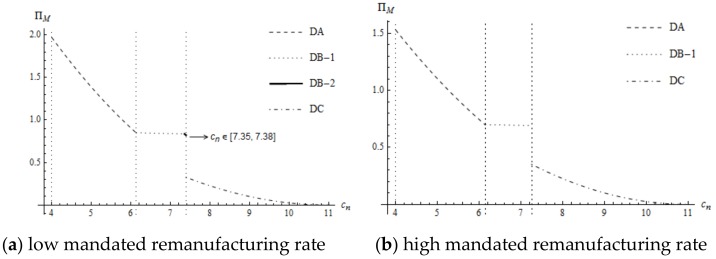
The impact of $c_{n}$ on the manufacturer’s profit.

Noteworthy, the supplier’s profit is continuously decreasing but there exists a piecewise point for the manufacturer’s profit. Whether high mandated remanufacturing target or low mandated remanufacturing target, once the optimal decision changes from DB-1 or DB-2 to DC, the profit of the manufacturer decreases rapidly. The rationale behind this is that the supplier’s wholesale price increases substantially and the quantity of the new product decreases rapidly. Although the quantity of remanufactured products increases, the decrease in profit due to new products exceeds the increase in profit due to the remanufactured products. Therefore, the manufacturer’s profit decreases substantially from DB-1 or DB-2 to DC, as are shown in Figure 5.

## 5. Impact of Mandated Remanufacturing Target on the Supply Chain’s Decision and Consumer Surplus

The government sets the lowest mandated remanufacturing target and requires manufacturers holding responsible for remanufacturing used products, aiming to improve the utilization of renewable resources as much as possible and incentive more environmental-friendly product design. In the paper, under the Decision DA and Decision DB-1, the mandated remanufacturing target is effective. Therefore, this section emphasizes the impact of the mandated remanufacturing target on the supply chain member’s optimal decisions under Decision DA and Decision DB-1, and thus explore whether the mandated remanufacturing target can bring more positive impacts and realize the government’s expectation. In addition, from the perspective of the consumer, we still analyze the impact of the mandated remanufacturing target on consumer surplus.

### 5.1. The Impact of Mandated Remanufacturing Target on the Manufacturer’s Decisions

In this part, we mainly investigate the effects of the mandated remanufacturing target on the manufacturer’s production decision and optimal profit.

Proposition 7. The effects of the mandated remanufacturing target on the manufacturer’s production decision and optimal profit are shown in Table 8.

For proof, see Appendix A.7.

The quantity of new product is decreasing in the mandated remanufacturing target, whereas the quantity of remanufactured product is increasing in the mandated remanufacturing target. The increase in mandated remanufacturing target demonstrates that the government’s requirement for remanufacturing becomes stricter, therefore there is no surprise that the quantity of remanufactured product increases. The rationale behind the decrease in the quantity of new product is that, when the remanufacturing cost advantage is not obvious, the manufacturer is unwilling to burden more responsibilities for remanufacturing, thereby decreasing the demands for new products to control the quantity of remanufactured product. The profit loss due to the decrease in the quantity of new product exceeds the profit due to the increase in the quantity of remanufactured product, thus the manufacturer’s profit is decreasing in the mandated remanufacturing target.

### 5.2. The Impact of Mandated Remanufacturing Target on the Supplier’s Decisions

This subsection mainly studies the effects of the mandated remanufacturing target on the supplier’s wholesale price, product design and optimal profit.

Proposition 8. The effects of mandated remanufacturing target on the supplier’s wholesale price, product design and optimal profit are shown in Table 9.

For proof, see Appendix A.8.

Proposition 8 indicates that the supplier’s wholesale price is increasing and its profit is decreasing in the mandated remanufacturing target under Decision DA and Decision DB-1. According to Proposition 7, as the mandated remanufacturing target improves, the quantity of new product decreases continuously, but the quantity of remanufactured product increases all the time. Therefore, the supplier wants to retain profits by improving the wholesale price. Because of the decrease in the quantity of new product, the supplier’s profit is decreasing in the mandated remanufacturing target.

Note, under Decision DA, with the increase in the mandated remanufacturing target, the supplier provides product design that is beneficial to remanufacturing and the used components are easier to be remanufactured. This is because that, the cost of the new component is low enough and the remanufacturing cost advantage does not exist under Decision DA. If the used components are difficult to be remanufactured, the manufacturer has to reduce demands for new components in order to meet the government’s requirement. Therefore, the supplier is forced to improve the level of interchangeability in new components to expand the manufacturer’s demand for the new component. In conclusion, under Decision DA, the increase in the mandated remanufacturing target can urge the supplier to implement product design that is beneficial to remanufacturing.

Compared with that under Decision DA, the remanufacturing cost advantage under Decision DB-1 is a little obvious. The impact of the mandated remanufacturing target on the supplier’s product design is also affected by the degree of acceptance of the remanufactured product. When the degree of acceptance of the remanufactured product is relatively high and the mandated remanufacturing target is too high, namely, $\alpha >\frac{1}{2}$ and $\frac{\sqrt{8\alpha -7\alpha^{2}}-\alpha }{2\alpha }<\gamma <1$, more stringent mandated remanufacturing target may cause an unexpected outcome. At this time, the higher mandated remanufacturing target cannot incentivize the supplier to provide more environmental-friendly product design. This is because, when the remanufacturing cost advantage improves, too high mandated remanufacturing target results in more remanufactured components competing with new components. Hence, the supplier reduces the level of interchangeability in new components to deter the manufacturer from remanufacturing. Under other conditions, with the increase in the mandated remanufacturing target, the supplier improves product design to make used components easier to be remanufactured, thereby enhancing the manufacturer’s demands for new components.

### 5.3. The Impact of the Mandated Remanufacturing Target on the Consumer Surplus

This part mainly explores the effects of the mandated remanufacturing target on the consumer surplus under Decision DA and Decision DB-1.

Proposition 9. Under Decision DA and Decision DB-1, as the mandated remanufacturing target increases, the consumer surplus decreases continually.

Based on proposition 8, we deduce that the price of the new product is increasing in the mandated remanufacturing target whereas the price of a remanufactured product is decreasing. The decrease in consumer surplus due to the increase in the price of a new product exceeds the increase in consumer surplus due to the decrease in the price of a remanufactured product. Therefore, the consumer surplus is decreasing in the mandated remanufacturing target.

## 6. Environmental Implications of Mandated Remanufacturing Target

The goal of the mandated remanufacturing target by the government is to reduce the negative environmental impacts of products. Compared with new products, remanufactured products are recognized to be more environmentally friendly, because remanufactured products consume less energy and raw materials. However, the increase in the amount of remanufactured products may lead to an undesirable environmental outcome. Therefore, we calculate the total environmental impacts of new and remanufactured products by using LCA approach and then identify the total environmental impacts of the mandated remanufacturing target.

LCA is a method that measures the product’s environmental impact from the perspective of the whole life cycle. From the perspective of the whole life cycle, the environmental impact of a product mainly comes from manufacturing a new product, remanufacturing, manufacturer’s proper disposal and customer usage and disposal. Considering specific contents in this paper, we assume that the environmental impact of a product comes from manufacturing a new product, remanufacturing and customer usage and disposal. In addition, we incorporate the environmental impact of supplier’s manufacturing a new component into that of manufacturing a new product. The environmental impact of manufacturing a new product $E_{n}$ comes from material extraction, processing, transportation and manufacturing. The environmental impact of remanufacturing $E_{r}$ comes from the collection of used products, disassembly, testing, processing, transportation and remanufacturing. The environmental impact of customer usage $E_{u}$ comprises of using new and remanufactured products. Similar to the literature [3], we consider that the environmental impact of using a new product is the same as that of using a remanufactured product. The environmental impact of customer disposal $E_{cd}$ originates from disposing of unreturned products (including new and remanufactured products) through landfill and incineration. Esenduran et al. consider that the environmental impacts of disposing new of and remanufactured products are the same [3]. Finally, we use E to represent total environmental impacts based on the LCA approach, that is $E=E_{n}+E_{r}+E_{u}+E_{cd}$. Let $\xi_{n}$, $\xi_{r}$, $\xi_{cd}$ and $\xi_{u}$ represent the unit environmental impact of a product in each stage. The environmental impact in each stage of the product life cycle can be represented as per unit environmental impact at the stage multiplying total quantities in the phase. Then, the environmental impact of each stage is respectively shown in Table 10. 

To facilitate our analysis, we define $x=\frac{\xi_{r}}{\xi_{n}}$, $y=\frac{\xi_{cd}}{\xi_{n}}$ and $z=\frac{\xi_{u}}{\xi_{n}}$. The above ratios represent the relative environmental impact of remanufacturing, customer usage and customer disposal compared to that of manufacturing new products, respectively. Currently, some literature considers that compared with new products, remanufactured products reduce the demands for energy and raw materials, bringing more positive environmental impacts. Thus, we assume that x≤1. In addition, consumers always dispose of unreturned products through landfill and incineration. Thus, the environmental impact of customer disposal is more likely to be higher than that of remanufacturing. Thus, we assume that x≤y. 

Proposition 10. Under decision DA, if $z\geq z_{1}$, then the environmental impact is increasing in the mandated remanufacturing target. Otherwise, it is decreasing in the mandated remanufacturing target; Under decision DB-1, if $z\geq z_{2}$, then the environmental impact is increasing in the mandated remanufacturing target. Otherwise, it is decreasing in the mandated remanufacturing target. In addition, $z_{1}$ and $z_{2}$, are increasing in the mandated remanufacturing target.

For proof, see Appendix A.9.

Proposition 10 indicates that mandated remanufacturing target cannot always bring positive impacts on the environment. The environmental implication of mandated remanufacturing target is closely related to the environmental impact of customer usage. Under these two decisions, if the environmental impact of customer usage is relatively low, namely $z<z_{1}$ and $z<z_{2}$, the government can improve environmental performance by increasing mandated remanufacturing target. If the environmental impact of customer usage is relatively high, namely $z\geq z_{1}$ and $z\geq z_{2}$, increasing mandated remanufacturing target contrarily results in worse environmental impact. In addition, $z_{1}$ and $z_{2}$ are increasing in the mandated remanufacturing target γ, respectively. Higher mandated remanufacturing target improves the threshold points of environmental impact in customer usage stage, which is beneficial to the environment. Based on the proposition 10, we find that setting mandated remanufacturing target alone is not enough to realize the government’s expectation of reducing pollution on the environment. The government still needs to guide customers to use products in an energy-efficient way in order to reduce the environmental impact of customer usage.

## 7. Conclusions

Along with the higher replacing frequency of electrical and electronic equipment, the number of WEEE becomes the fastest growing waste stream. Traditional treatments of WEEE, such as landfill and incineration, cause harm to the environment and resources. In order to properly deal with the WEEE, many countries in the world have implemented various forms of take back legislation, which aims to reduce pollution to the environment, waste of resource and incentivize product design. Among them, a recent recast of WEEE has put forward that a specific reuse target should be considered. In our paper, we construct a Stackelberg game model to describe a two-echelon closed-loop supply chain consisting of a qualified EEE component supplier (the leader) and a qualified EEE manufacturer (the follower) with the mandated remanufacturing target. We characterize the supplier’s optimal product design and wholesale pricing, and the manufacturer’s optimal production decisions. Then, we analyze the impact of the main parameters on the supplier’s and the manufacturer’s optimal decisions and consumer surplus. Especially, we emphasize the impact of the mandated remanufacturing target on the supplier’s product design in order to examine whether the goal of incentivizing green product design can be achieved. At last, we investigate the environmental implication of mandated remanufacturing target, in order to examine whether the goal of reducing pollution can be fulfilled. The main conclusions are as follows:

First, the mandated remanufacturing target affects the supplier’s and the manufacturer’s optimal decisions. Over stringent mandated remanufacturing target, namely $\gamma^{*}\leq \gamma <1$, leads that there is no chance for the manufacturer to remanufacture part of used products voluntarily.

Secondly, the optimal decisions of the supplier and the manufacturer are affected by each other’s cost parameters. The impacts of the cost of the new component, the cost of remanufacturing and the remanufacturing saving cost due to product design are different based on the supplier’s responsibility of product design and the manufacturer’s responsibility of remanufacturing.

Thirdly, we show that mandated remanufacturing target is harmful to economic profits of the supply chain’s members and consumer surplus. Although mandated remanufacturing target results in the increase in the quantity of remanufactured product, it also results in a decrease in the quantity of new product. Thus, the economic profits of the supplier and the manufacturer and the consumer surplus are decreasing.

Fourthly, the impact of the mandated remanufacturing target on the supplier’s product design is more complex. We find that mandated remanufacturing target cannot always incentivize the supplier to provide product design that is beneficial to remanufacturing. When the consumer’s recognition for remanufactured products is relatively high and mandated remanufacturing target is too high, increasing the mandated remanufacturing target contrarily results in the decrease in the level of interchangeability in product design.

Finally, although remanufactured products are more environmentally friendly than new products, a more stringent mandated remanufacturing target cannot ensure a better environmental impact. The finding is that the environmental implication of mandated remanufacturing target is closely related with the environmental impact of customer usage. If the environmental impact of customer usage is too high, namely, $\alpha >\frac{1}{2}$ and $\frac{\sqrt{8\alpha -7\alpha^{2}}-\alpha }{2\alpha }<\gamma <1$, the increase in the mandated remanufacturing target will bring worse environmental impact. This reminds us that the policymakers should advocate and educate customers to use products in an energy efficient way instead of only improving mandated remanufacturing target.

There are still some limitations to our paper. First, we do not consider the competitions from remanufacturers; second, we only take remanufacturing into account and ignore other ways of reuses, such as recycling. Therefore, we can further take competition from remanufacturers into account and consider recycling and other methods of reuse.

## Figures and Tables

**Table 1:** Notations.

Notations	Description
Variables	
$q_{n},p_{n}$	Quantity and price of the new product
$q_{r},p_{r}$	Quantity and price of the remanufactured product
w	Wholesale price of the new component
ω	The level of interchangeability in the new component
Parameters	
$c_{n}$	Unit cost of the new component
$c_{r}$	Unit cost of the remanufactured component
κ	Scaling parameter of product design
Δ	Unit saving cost due to product design
α	The degree of acceptance of the remanufactured product
γ	Mandated remanufacturing target

**Table 2:** The supplier’s optimal wholesale price and product design.

Decision	w	ω
SA	$\begin{array}{l} Q+\frac{2\kappa \rho_{g}\left(1+\alpha \rho_{g}\left(2+\rho_{g}\right)\right)H_{2}}{4\kappa +8\alpha \kappa \rho_{g}+\left(4\alpha \kappa -\Delta^{2}\right)\rho_{g}^{2}}\\ -\frac{\left(2\kappa \left(1+\alpha \rho_{g}\left(2+\rho_{g}\right)\right)-\Delta^{2}\rho_{g}^{2}\right)H_{1}}{4\kappa +8\alpha \kappa \rho_{g}+\left(4\alpha \kappa -\Delta^{2}\right)\rho_{g}^{2}} \end{array}$	$\frac{\Delta \rho_{g}\left(H_{1}+H_{2}\rho_{g}\right)}{4\kappa +8\alpha \kappa \rho_{g}+\left(4\alpha \kappa -\Delta^{2}\right)\rho_{g}^{2}}$
SB-1	$Q-\frac{2\alpha \kappa \left(1+\rho_{g}\right)\left(1+\alpha \rho_{g}\right)H_{2}+\left(1+\alpha \rho_{g}\right)\Delta^{2}H_{1}}{2\left(\Delta^{2}+\alpha^{2}\kappa +\alpha \rho_{g}\left(\Delta^{2}+2\alpha \kappa +\alpha \kappa \rho_{g}\right)\right)}$	$\frac{\Delta \left(\alpha H_{1}\left(1+\rho_{g}\right)-2H_{2}\left(1+\alpha \rho_{g}\right)\right)}{2\left(\Delta^{2}+\alpha^{2}\kappa +\alpha \rho_{g}\left(\Delta^{2}+2\alpha \kappa +\alpha \kappa \rho_{g}\right)\right)}$
SB-2	$Q-\frac{\left(2\left(1-\alpha \right)\kappa -\Delta^{2}\right)H_{1}+2\left(1-\alpha \right)\kappa H_{2}}{4\left(1-\alpha \right)\kappa -\Delta^{2}}$	$-\frac{\Delta \left(H_{1}-H_{2}\right)}{4\left(1-\alpha \right)\kappa -\Delta^{2}}$
SC	$Q-\frac{H_{1}\left(\left(1+3\alpha \right)\kappa -\Delta^{2}\right)-2H_{2}\left(1+3\alpha \right)\kappa }{4\left(\kappa +3\alpha \kappa \right)-\Delta^{2}}$	$\frac{\Delta \left(H_{1}+H_{2}\right)}{4\left(\kappa +3\alpha \kappa \right)-\Delta^{2}}$

**Table 3:** The manufacturer’s optimal production decision.

Manufacturer’s Decision	$q_{n}$	$q_{r}$	Supplier’s Corresponding Decision
MA	$\frac{\kappa \left(H_{1}+H_{2}\gamma \right)}{4\kappa +8\alpha \kappa \gamma +\left(4\alpha \kappa -\Delta^{2}\right)\gamma^{2}}$	$\frac{\kappa \gamma \left(H_{1}+H_{2}\gamma \right)}{4\kappa +8\alpha \kappa \gamma +\left(4\alpha \kappa -\Delta^{2}\right)\gamma^{2}}$	SA
MB-1	$\frac{\Delta^{2}H_{1}+2H_{2}\alpha \kappa \left(1+\gamma \right)}{4\left(\Delta^{2}+\alpha^{2}\kappa +\alpha \left(\Delta^{2}+2\alpha \kappa \right)\gamma +\alpha^{2}\kappa \gamma^{2}\right)}$	$\frac{\gamma \left(\Delta^{2}H_{1}+2H_{2}\alpha \kappa \left(1+\gamma \right)\right)}{4\left(\Delta^{2}+\alpha^{2}\kappa +\alpha \left(\Delta^{2}+2\alpha \kappa \right)\gamma +\alpha^{2}\kappa \gamma^{2}\right)}$	SB-1
MB-2	$\frac{\kappa \left(H_{1}-H_{2}\right)}{4\left(1-\alpha \right)\kappa -\Delta^{2}}$	$\frac{2\left(2-\alpha \right)\kappa H_{2}-H_{1}\left(\Delta^{2}+2\alpha \kappa \right)}{2\alpha \left(4\left(1-\alpha \right)\kappa -\Delta^{2}\right)}$	SB-2
MC	$\frac{\kappa \left(H_{1}+H_{2}\right)}{4\left(\kappa +3\alpha \kappa \right)-\Delta^{2}}$	$\frac{\kappa \left(H_{1}+H_{2}\right)}{4\left(\kappa +3\alpha \kappa \right)-\Delta^{2}}$	SC

**Table 4:** The impacts of cost parameters on the supplier’s product design.

Parameter	$\omega^{DA}$	$\omega^{DB-1}$	$\omega^{DB-2}$	$\omega^{DC}$
$c_{n}$	−	−	+	−
$c_{r}$	−	+	−	−
Δ	+	$\pm^{*}$	−	+

* if $c_{n}>c_{n1}$, −; otherwise, +.

**Table 5:** Sensitivity analysis of the supplier’s optimal wholesale prices.

Parameter	*w^DA^*	*w^DB−1^*	*w^DB−2^*	*w^DC^*
$c_{n}$	+	+	+	+
$c_{r}$	−	+	+	−
Δ	+	± *	+	+

* if $c_{n}>c_{n1}$, −; otherwise, +.

**Table 6:** Sensitivity analysis of the manufacturer’s optimal production decisions.

Parameter	$q_{n}^{DA}$	$q_{n}^{DB-1}$	$q_{n}^{DB-2}$	$q_{n}^{DC}$	$q_{r}^{DA}$	$q_{r}^{DB-1}$	$q_{r}^{DB-2}$	$q_{r}^{DC}$
$c_{n}$	−	−	−	−	−	−	+	−
$c_{r}$	−	−	+	−	−	−	−	−
Δ	+	$\pm^{*}$	+	+	+	$\pm^{*}$	−	+

* if $c_{n}>c_{n1}$, −; otherwise, +.

**Table 7:** Sensitivity analysis of the supplier’s and the manufacturer’s optimal profits.

Parameter	$\Pi_{S}^{DA}$	$\Pi_{S}^{DB-1}$	$\Pi_{S}^{DB-2}$	$\Pi_{S}^{DC}$	$\Pi_{M}^{DA}$	$\Pi_{M}^{DB-1}$	$\Pi_{M}^{DB-2}$	$\Pi_{M}^{DC}$
$c_{n}$	−	−	−	−	−	−	−	−
$c_{r}$	−	$\mp^{**}$	+	−	−	−	−	−
Δ	+	+	+	+	+	$\pm^{*}$	−	+

* if $c_{n}>c_{n1}$, −; otherwise, +. ** if $c_{n}>c_{n1}$, +; otherwise, −.

**Table 8:** The impact of the mandated remanufacturing target on the manufacturer’s decisions.

Parameter	$q_{n}^{DA}$	$q_{n}^{DB-1}$	$q_{r}^{DA}$	$q_{r}^{DB-1}$	$\Pi_{M}^{DA}$	$\Pi_{M}^{DB-1}$
γ	−	−	+	+	−	−

**Table 9:** The impact of mandated remanufacturing target on the supplier’s decisions.

Parameter	$w^{DA}$	$w^{DB-1}$	$\omega^{DA}$	$\omega^{DB-1}$	$\Pi_{S}^{DA}$	$\Pi_{S}^{DB-1}$
γ	+	+	+	$+{\left(\mp \right)}^{***}$	−	−

*** When $\alpha >\frac{1}{2}$ and $0<\gamma <\frac{\sqrt{8\alpha -7\alpha^{2}}-\alpha }{2\alpha }$ or $\alpha \leq \frac{1}{2}$, + in $c_{n}\in (A,K_{4}(B_{1}))$. When $\alpha >\frac{1}{2}$ and $\frac{\sqrt{8\alpha -7\alpha^{2}}-\alpha }{2\alpha }<\gamma <1$,− in $c_{n}\in (A,c_{n2})$; + in $c_{n}\in (c_{n2},K_{4}(B_{1}))$. Here, $c_{n2}=\frac{\alpha \kappa \left(1+\rho_{g}\right)\left(Q\alpha \left(1+\rho_{g}\right)-2\left(Q\alpha -c_{r}\right)\left(2-\alpha +\alpha \rho_{g}\right)\right)-Q\left(1-\alpha \right)\Delta^{2}}{\alpha^{2}\kappa \left(1+\rho_{g}\left(2+\rho_{g}\right)\right)-\left(1-\alpha \right)\Delta^{2}}$.

**Table 10:** Environmental Impact Expression.

Phase	Environmental Impact	Phase	Environmental Impact
Manufacturer		Customer	
Manufacturing	$E_{n}=q_{n}\xi_{n}$	Customer disposal	$E_{cd}=\left(q_{n}-q_{r}+q_{r}\right)\xi_{cd}$
Remanufacturing	$E_{r}=q_{r}\xi_{r}$	Customer usage	$E_{u}=\left(q_{n}+q_{r}\right)\xi_{u}$

## Appendix A

### Appendix A.1

The model is $\Pi_{M}\left(q_{n},q_{r}\right)=q_{n}\left(p_{n}-w\right)+q_{r}\left(p_{r}-c_{r}+\Delta \omega \right)$ subject to $\gamma q_{n}\leq q_{r}\leq q_{n}$. The Hessian matrix of $\Pi_{M}$ is negative definite and the profit function of $\Pi_{M}$ is concave of $q_{n}$ and $q_{r}$. Solving KKT conditions $\frac{\partial \Pi_{M}}{\partial q_{n}}=Q-w-2q_{n}-2\alpha q_{r}+\lambda_{1}-\lambda_{2}\gamma =0$, $\frac{\partial \Pi_{M}}{\partial q_{r}}=\Delta \omega -c_{r}-\alpha q_{n}+\alpha \left(Q-q_{n}-q_{r}\right)-\alpha q_{r}-\lambda_{1}+\lambda_{2}=0$, $\lambda_{1}\left(q_{n}-q_{r}\right)=0$ and $\lambda_{2}\left(q_{r}-\gamma q_{n}\right)=0$, we can get three feasible decisions according to Complementary Slackness Theorem.

Decision MA: $\lambda_{1}=0$ and $\lambda_{2}>0$.

Solving $q_{r}=\gamma q_{n}$ and the first order conditions give $\lambda_{2}=\frac{-w\alpha -\Delta \omega +c_{r}+\alpha \left(Q-w-Q\alpha -\Delta \omega +c_{r}\right)\gamma }{1+\alpha \lambda \left(2+\gamma \right)}$, $q_{n}^{MA}=\frac{Q-w+Q\alpha \gamma +\Delta \omega \gamma -c_{r}\gamma }{2\left(1+2\alpha \gamma +\alpha \gamma^{2}\right)}$ and $q_{r}^{MA}=\gamma q_{n}^{MA}$. $\lambda_{2}>0$ and $0<\gamma q_{n}\leq q_{r}\leq q_{n}$ give $w<\frac{-\Delta \omega +c_{r}+\alpha \left(Q-Q\alpha -\Delta \omega +c_{r}\right)\gamma }{\alpha \left(1+\gamma \right)}$ and $w\leq Q+\left(Q\alpha +\Delta \omega -c_{r}\right)\gamma$. $\frac{-\Delta \omega +c_{r}+\alpha \left(Q-Q\alpha -\Delta \omega +c_{r}\right)\gamma }{\alpha \left(1+\gamma \right)}<Q+\left(Q\alpha +\Delta \omega -c_{r}\right)\gamma$. Therefore, Decision MA exists when $w<\frac{-\Delta \omega +c_{r}+\alpha \left(Q-Q\alpha -\Delta \omega +c_{r}\right)\gamma }{\alpha \left(1+\gamma \right)}$.

Decision MB: $\lambda_{1}=0$ and $\lambda_{2}=0$.

Solving the first order conditions gives $q_{n}^{MB}=\frac{Q-w-Q\alpha -\Delta \omega +c_{r}}{2\left(1-\alpha \right)}$ and $q_{r}^{MB}=\frac{w\alpha +\Delta \omega -c_{r}}{2\left(1-\alpha \right)\alpha }$. $0<\gamma q_{n}\leq q_{r}\leq q_{n}$ gives $w\leq Q-Q\alpha -\Delta \omega +c_{r}$, $w\leq \frac{Q\alpha -Q\alpha^{2}-\Delta \omega -\alpha \Delta \omega +c_{r}+\alpha c_{r}}{2\alpha }$, $w\geq \frac{-\Delta \omega +c_{r}}{\alpha }$ and $w\geq \frac{-\Delta \omega +c_{r}+\alpha \left(Q-Q\alpha -\Delta \omega +c_{r}\right)\gamma }{\alpha \left(1+\gamma \right)}$, respectively. $\frac{Q\alpha -Q\alpha^{2}-\Delta \omega -\alpha \Delta \omega +c_{r}+\alpha c_{r}}{2\alpha }<Q-Q\alpha -\Delta \omega +c_{r}$. $\frac{-\Delta \omega +c_{r}+\alpha \left(Q-Q\alpha -\Delta \omega +c_{r}\right)\gamma }{\alpha \left(1+\gamma \right)}>\frac{-\Delta \omega +c_{r}}{\alpha }$. Therefore, Decision MB exists when $\frac{-\Delta \omega +c_{r}+\alpha \left(Q-Q\alpha -\Delta \omega +c_{r}\right)\gamma }{\alpha \left(1+\gamma \right)}\leq w\leq \frac{Q\alpha -Q\alpha^{2}-\Delta \omega -\alpha \Delta \omega +c_{r}+\alpha c_{r}}{2\alpha }$.

Decision MC: $\lambda_{1}>0$ and $\lambda_{2}=0$.

Solving $q_{r}=q_{n}$ and the first order conditions give $\lambda_{1}=\frac{2w\alpha +Q\left(-1+\alpha \right)\alpha +\left(1+\alpha \right)\Delta \omega -\left(1+\alpha \right)c_{r}}{1+3\alpha }$, $q_{n}^{MC}=\frac{Q-w+Q\alpha +\Delta \omega -c_{r}}{2\left(1+3\alpha \right)}$ and $q_{r}^{MC}=q_{n}^{MC}$. $0<\gamma q_{n}\leq q_{r}\leq q_{n}$ and $\lambda_{1}>0$ give $w>\frac{Q\alpha -Q\alpha^{2}-\Delta \omega -\alpha \Delta \omega +c_{r}+\alpha c_{r}}{2\alpha }$ and $w\leq Q+Q\alpha +\Delta \omega -c_{r}$, respectively. Therefore, Decision MC exists when $\frac{Q\alpha -Q\alpha^{2}-\Delta \omega -\alpha \Delta \omega +c_{r}+\alpha c_{r}}{2\alpha }<w\leq Q+Q\alpha +\Delta \omega -c_{r}$.

### Appendix A.2

The supplier’s profit function is $\Pi_{S}\left(w,\omega \right)=\left(w-c_{n}\right)q_{n}-\frac{1}{2}\kappa \omega^{2}$.

Let $H_{1}=Q-c_{n}$, $H_{2}=Q\alpha -c_{r}$ and $H_{3}=\alpha c_{n}-c_{r}$. $H_{1}>H_{2}$.

Decision SA:

When the supplier anticipates that the manufacturer will adopt decision MA, then the corresponding profit function of the supplier is $\Pi_{S}^{SA}\left(w^{SA},\omega^{SA}\right)=\left(w^{SA}-c_{n}\right)q_{n}^{MA}-\frac{1}{2}\kappa {\left(\omega^{SA}\right)}^{2}$, which subjects to $c_{n}<w^{SA}<\frac{-\Delta \omega +c_{r}+\alpha \left(Q-Q\alpha -\Delta \omega +c_{r}\right)\gamma }{\alpha \left(1+\gamma \right)}$. The Hessian matrix is negative definite and the profit function is concave. Solving KKT conditions $\frac{\partial \Pi_{S}^{SA}}{\partial w^{SA}}=\lambda_{1}-\lambda_{2}-\frac{w^{SA}-c_{n}}{2+2\alpha \gamma \left(2+\gamma \right)}+\frac{Q-w^{SA}+\left(Q\alpha +\Delta \omega^{SA}-c_{r}\right)\gamma }{2+2\alpha \gamma \left(2+\gamma \right)}=0$, $\frac{\partial \Pi_{S}^{SA}}{\partial \omega^{SA}}=-\kappa \omega^{SA}+\frac{\lambda_{2}\left(-\Delta -\alpha \Delta \gamma \right)}{\alpha \left(1+\gamma \right)}+\frac{\Delta \left(w^{SA}-c_{n}\right)\gamma }{2+2\alpha \gamma \left(2+\gamma \right)}=0$, $\mu_{1}\left(w^{SA}-c_{n}\right)=0$ and $\mu_{2}\left(\frac{-\Delta \omega^{SA}+c_{r}+\alpha \left(Q-Q\alpha -\Delta \omega^{SA}+c_{r}\right)\gamma }{\alpha \left(1+\gamma \right)}-w^{SA}\right)=0$, we get one feasible decision according to Complementary Slackness Theorem.

$\mu_{1}=0$ and $\mu_{2}=0$. Solving the first order conditions gives $w^{SA}=\frac{\left(\Delta^{2}\gamma^{2}-2\kappa \left(1+\alpha \gamma \left(2+\gamma \right)\right)\right)H_{1}+2\kappa H_{2}\gamma \left(1+\alpha \gamma \left(2+\gamma \right)\right)}{4\kappa +8\alpha \kappa \gamma -\left(\Delta^{2}-4\alpha \kappa \right)\gamma^{2}}+Q$ and $\omega^{SA}=\frac{\Delta \gamma \left(H_{1}+H_{2}\gamma \right)}{4\kappa +8\alpha \kappa \gamma -\left(\Delta^{2}-4\alpha \kappa \right)\gamma^{2}}$. $w^{SA}-c_{n}\geq 0$ and $\frac{-\Delta \omega +c_{r}+\alpha \left(Q-Q\alpha -\Delta \omega +c_{r}\right)\gamma }{\alpha \left(1+\gamma \right)}-w^{SA}\geq 0$ give $c_{n}\leq Q+Q\alpha \gamma -c_{r}\gamma$ and $c_{n}\leq A=\frac{2\kappa c_{r}\left(2+\alpha \gamma \left(3+\gamma \right)\right)+Q\left(-2\alpha \kappa -\gamma \left(\Delta^{2}+2\alpha \left(-1+3\alpha \right)\kappa +2\alpha^{2}\kappa \gamma \right)\right)}{2\alpha \kappa -\left(\Delta^{2}-2\alpha \kappa \right)\gamma }$, respectively. $A<Q+Q\alpha \gamma -c_{r}\gamma$. Therefore, Decision S-A exists when $c_{r}<c_{n}\leq A$.

Decision SB:

When the supplier anticipates that the manufacturer will adopt decision MB, then the corresponding profit function of the supplier is $\Pi_{S}^{SB}\left(w^{SB},\omega^{SB}\right)=\left(w^{SB}-c_{n}\right)q_{n}^{MB}-\frac{1}{2}\kappa {\left(\omega^{SB}\right)}^{2}$, which subjects to $\frac{-\Delta \omega +c_{r}+\alpha \left(Q-Q\alpha -\Delta \omega +c_{r}\right)\gamma }{\alpha \left(1+\gamma \right)}\leq w^{SB}\leq \frac{Q\alpha -Q\alpha^{2}-\Delta \omega -\alpha \Delta \omega +c_{r}+\alpha c_{r}}{2\alpha }$. The Hessian matrix is negative definite and the profit function is concave. Solving the KKT conditions $\frac{\partial \Pi_{S}^{SB}}{\partial w^{SB}}=\frac{1}{2}\left(\frac{w^{SB}-c_{n}}{-1+\alpha }+\frac{w^{SB}+Q\left(-1+\alpha \right)+\Delta \omega^{SB}-c_{r}}{-1+\alpha }\right)+\mu_{3}-\mu_{4}=0$, $\frac{\partial \Pi_{S}^{SB}}{\partial \omega^{SB}}=\frac{1}{2}\left(-2\kappa \omega^{SB}+\frac{\Delta \left(w^{SB}-c_{n}\right)}{-1+\alpha }\right)+\frac{\left(-\Delta -\alpha \Delta \right)\mu_{4}}{2\alpha }-\frac{\mu_{3}\left(-\Delta -\alpha \Delta \gamma \right)}{\alpha \left(1+\gamma \right)}=0$,$\mu_{3}\left(w^{SB}-\frac{-\Delta \omega^{SB}+c_{r}+\alpha \left(Q-Q\alpha -\Delta \omega^{SB}+c_{r}\right)\gamma }{\alpha \left(1+\gamma \right)}\right)=0$ and $\mu_{4}\left(\frac{Q\alpha -Q\alpha^{2}-\Delta \omega^{SB}-\alpha \Delta \omega^{SB}+c_{r}+\alpha c_{r}}{2\alpha }-w^{SB}\right)=0$, we get three feasible decisions according to Complementary Slackness Theorem.

Decision SB-1: $\mu_{3}>0$ and $\mu_{4}=0$.

Solving the first order conditions gives $w^{SB-1}=\frac{-H_{2}\left(2\alpha \kappa \left(1+\gamma \right)\left(1+\alpha \gamma \right)\right)-\Delta^{2}H_{1}\left(1+\alpha \gamma \right)}{2\left(\Delta^{2}+\alpha^{2}\kappa +\alpha \gamma \left(\Delta^{2}+2\alpha \kappa +\alpha \kappa \gamma \right)\right)}+Q$, $\omega^{SB-1}=\frac{\Delta \left(\alpha H_{1}\left(1+\gamma \right)-2H_{2}\left(1+\alpha \gamma \right)\right)}{2\left(\Delta^{2}+\alpha^{2}\kappa +\alpha \gamma \left(\Delta^{2}+2\alpha \kappa +\alpha \kappa \gamma \right)\right)}$ and $\mu_{3}=\frac{\alpha \left(1+\gamma \right)(-Q\left(\Delta^{2}+2\left(-1+\alpha \right)\alpha \kappa \right)+2\left(-2+\alpha \right)\kappa c_{r}-2\alpha \kappa \left(Q-Q\alpha +c_{r}\right)\gamma +c_{n}\left(\Delta^{2}+2\alpha \kappa +2\alpha \kappa \gamma \right))}{4\left(-1+\alpha \right)\left(\Delta^{2}+\alpha^{2}\kappa +\alpha \gamma \left(\Delta^{2}+2\alpha \kappa +\alpha \kappa \gamma \right)\right)}$. $\mu_{3}>0$ and $w^{SB}-\frac{-\Delta \omega^{SB}+c_{r}+\alpha \left(Q-Q\alpha -\Delta \omega^{SB}+c_{r}\right)\gamma }{\alpha \left(1+\gamma \right)}\geq 0$ give $c_{n}<B_{1}=\frac{Q\left(\Delta^{2}+2\left(-1+\alpha \right)\alpha \kappa \right)-2\left(-2+\alpha \right)\kappa c_{r}+2\alpha \kappa \left(Q-Q\alpha +c_{r}\right)\gamma }{\Delta^{2}+2\alpha \kappa +2\alpha \kappa \gamma }$ and $c_{n}\leq \frac{-2\alpha \kappa c_{r}\left(1+\gamma \right)+Q\left(\Delta^{2}+2\alpha^{2}\kappa +2\alpha^{2}\kappa \gamma \right)}{\Delta^{2}}$, respectively. $B_{1}<\frac{-2\alpha \kappa c_{r}\left(1+\gamma \right)+Q\left(\Delta^{2}+2\alpha^{2}\kappa +2\alpha^{2}\kappa \gamma \right)}{\Delta^{2}}$. Therefore, Decision SB-1 exists when $c_{r}<c_{n}<B_{1}$.

Decision SB-2: $\mu_{3}=0$ and $\mu_{4}=0$.

Solving the first order conditions gives $w^{SB-2}=\frac{H_{1}\left(-\Delta^{2}+2\left(1-\alpha \right)\kappa \right)+2\left(1-\alpha \right)\kappa H_{2}}{\Delta^{2}+4\left(-1+\alpha \right)\kappa }+Q$ and $\omega^{SB-2}=\frac{\Delta \left(H_{1}-H_{2}\right)}{\Delta^{2}-4\kappa +4\alpha \kappa }$. $w^{SB}-\frac{-\Delta \omega^{SB}+c_{r}+\alpha \left(Q-Q\alpha -\Delta \omega^{SB}+c_{r}\right)\gamma }{\alpha \left(1+\gamma \right)}\geq 0$ and $\frac{Q\alpha -Q\alpha^{2}-\Delta \omega^{SB}-\alpha \Delta \omega^{SB}+c_{r}+\alpha c_{r}}{2\alpha }-w^{SB}\geq 0$ give $c_{n}\geq B_{1}$ and $c_{n}\leq B_{2}=\frac{Q\Delta^{2}+4\kappa c_{r}}{\Delta^{2}+4\alpha \kappa }$, respectively. $B_{1}<B_{2}$. Therefore, Decision SB-2 exists when $B_{1}\leq c_{n}\leq B_{2}$.

Decision SB-3: $\mu_{3}=0$ and

Solving the first order conditions gives $w^{SB-3}=Q-\frac{\left(1+\alpha \right)\Delta^{2}H_{1}+4\alpha \kappa \left(1+\alpha \right)H_{2}}{2\left(1+\alpha \right)\Delta^{2}+8\alpha^{2}\kappa }$, $\omega^{SB-3}=-\frac{\Delta \left(\alpha H_{2}+H_{3}\right)}{\Delta^{2}+\alpha \Delta^{2}+4\alpha^{2}\kappa }$ and $\mu_{4}=\frac{-Q\alpha \Delta^{2}+\alpha \Delta^{2}c_{n}+4\alpha^{2}\kappa c_{n}-4\alpha \kappa c_{r}}{2\left(1-\alpha \right)\left(\Delta^{2}+\alpha \Delta^{2}+4\alpha^{2}\kappa \right)}$. $\mu_{4}>0$ and $\frac{Q\alpha -Q\alpha^{2}-\Delta \omega^{SB}-\alpha \Delta \omega^{SB}+c_{r}+\alpha c_{r}}{2\alpha }-w^{SB}\geq 0$ give $c_{n}>B_{2}$ and $c_{n}\leq B_{3}=\frac{Q\Delta^{2}+4Q\alpha^{2}\kappa -4\alpha \kappa c_{r}}{\Delta^{2}}$, respectively. $B_{2}<B_{3}$. Therefore, Decision SB-3 exists when $B_{2}<c_{n}\leq B_{3}$.

Decision SC:

When the supplier anticipates that the manufacturer will adopt decision MC, then the corresponding profit function of the supplier is $\Pi_{S}^{SC}\left(w^{SC},\omega^{SC}\right)=\left(w^{SC}-c_{n}\right)q_{n}^{MC}-\frac{1}{2}\kappa {\left(\omega^{SC}\right)}^{2}$, which subjects to $\frac{Q\alpha -Q\alpha^{2}-\Delta \omega -\alpha \Delta \omega +c_{r}+\alpha c_{r}}{2\alpha }<w^{SC}\leq Q+Q\alpha +\Delta \omega -c_{r}$. The Hessian matrix is negative definite, and the profit function is concave. Solving the KKT conditions $\frac{\partial \Pi_{S}^{SC}}{\partial w^{SC}}=-\frac{w^{SC}-c_{n}}{2+6\alpha }+\frac{Q-w^{SC}+Q\alpha +\Delta \omega^{SC}-c_{r}}{2+6\alpha }+\mu_{5}-\mu_{6}=0$, $\frac{\partial \Pi_{S}^{SC}}{\partial \omega^{SC}}=-\kappa \omega^{SC}+\frac{\Delta \left(w^{SC}-c_{n}\right)}{2+6\alpha }-\frac{\left(-\Delta -\alpha \Delta \right)\mu_{5}}{2\alpha }+\Delta \mu_{6}=0$, $\mu_{5}\left(w^{SC}-\frac{Q\alpha -Q\alpha^{2}-\Delta \omega^{SC}-\alpha \Delta \omega^{SC}+c_{r}+\alpha c_{r}}{2\alpha }\right)=0$ and $\mu_{6}\left(Q+Q\alpha +\Delta \omega^{SC}-c_{r}-w^{SC}\right)=0$, we get one feasible decision according to Complementary Slackness Theorem.

$\mu_{5}=0$ and $\mu_{6}=0$. Solving the first order conditions gives $w^{SC}=\frac{-2\left(1+3\alpha \right)\kappa H_{2}+H_{1}\left(\left(1+3\alpha \right)\kappa -\Delta^{2}\right)}{\Delta^{2}-4\left(\kappa +3\alpha \kappa \right)}+Q$ and $\omega^{SC}=\frac{\Delta \left(H_{1}+H_{2}\right)}{-\Delta^{2}+4\left(\kappa +3\alpha \kappa \right)}$. $w^{SC}-\frac{Q\alpha -Q\alpha^{2}-\Delta \omega^{SC}-\alpha \Delta \omega^{SC}+c_{r}+\alpha c_{r}}{2\alpha }\geq 0$ and $Q+Q\alpha +\Delta \omega^{SC}-c_{r}-w^{SC}\geq 0$ give $c_{n}\geq C_{1}=\frac{Q\Delta^{2}+8Q\alpha^{2}\kappa -4\kappa c_{r}-8\alpha \kappa c_{r}}{\Delta^{2}-4\alpha \kappa }$ and $c_{n}\leq C_{2}=Q+Q\alpha -c_{r}$, respectively. $C_{1}<C_{2}$. Therefore, Decision S-C exists when $C_{1}\leq c_{n}\leq C_{2}$.

Substitute the supplier’s optimal interchangeable level of key component and wholesale price into the profits, then we can obtain the supplier’s and manufacturer’s profits (Table A1).

**Table A1:** The optimal profits.

Decision	$\Pi_{S}$	$\Pi_{M}$
DA	$\frac{\kappa {\left(H_{1}+H_{2}\gamma \right)}^{2}}{2\left(4\kappa +8\alpha \kappa \gamma -\left(\Delta^{2}-4\alpha \kappa \right)\gamma^{2}\right)}$	$\frac{\kappa^{2}{\left(H_{1}+H_{2}\gamma \right)}^{2}\left(1+2\alpha \gamma +\alpha \gamma^{2}\right)}{{\left(4\kappa +8\alpha \kappa \gamma -\left(\Delta^{2}-4\alpha \kappa \right)\gamma^{2}\right)}^{2}}$
DB-1	$Q-\frac{H_{2}\left(2\alpha \kappa \left(1+\gamma \right)\left(1+\alpha \gamma \right)\right)+\Delta^{2}H_{1}\left(1+\alpha \gamma \right)}{2\left(\Delta^{2}+\alpha^{2}\kappa +\alpha \gamma \left(\Delta^{2}+2\alpha \kappa +\alpha \kappa \gamma \right)\right)}$	$\frac{\left(1+2\alpha \gamma +\alpha \gamma^{2}\right){\left(\Delta^{2}H_{1}+2\alpha \kappa H_{2}\left(1+\gamma \right)\right)}^{2}}{16{\left(\Delta^{2}+\alpha^{2}\kappa +\alpha \left(\Delta^{2}+2\alpha \kappa \right)\gamma +\alpha^{2}\kappa \gamma^{2}\right)}^{2}}$
DB-2	$\frac{\kappa {\left(H_{2}-H_{1}\right)}^{2}}{2\left(-\Delta^{2}+4\left(1-\alpha \right)\kappa \right)}$	$-\frac{\Delta \left(H_{1}-H_{2}\right)}{4\left(1-\alpha \right)\kappa -\Delta^{2}}$
DB-3	$Q-\frac{\left(2\left(1-\alpha \right)\kappa -\Delta^{2}\right)H_{1}+2\left(1-\alpha \right)\kappa H_{2}}{4\left(1-\alpha \right)\kappa -\Delta^{2}}$	$\frac{\left(1+3\alpha \right){\left(\Delta^{2}H_{1}+4\alpha \kappa H_{2}\right)}^{2}}{16{\left(\left(1+\alpha \right)\Delta^{2}+4\alpha^{2}\kappa \right)}^{2}}$
DC	$\frac{\kappa {\left(H_{1}+H_{2}\right)}^{2}}{2\left(-\Delta^{2}+4\left(\kappa +3\alpha \kappa \right)\right)}$	$\frac{\left(1+3\alpha \right)\kappa^{2}{\left(H_{1}+H_{2}\right)}^{2}}{{\left(\Delta^{2}-4\left(\kappa +3\alpha \kappa \right)\right)}^{2}}$

Compare boundary points of each decision, the results are as follows:

When $0<\gamma <\frac{-4\Delta^{2}-3\alpha \Delta^{2}+4\alpha \kappa -12\alpha^{2}\kappa }{\alpha \left(\Delta^{2}+4\kappa +4\alpha \kappa \right)}$, then $c_{r}<A<B_{1}<C_{1}<B_{2}<C_{2}<B_{3}$.

When $\frac{-4\Delta^{2}-3\alpha \Delta^{2}+4\alpha \kappa -12\alpha^{2}\kappa }{\alpha \left(\Delta^{2}+4\kappa +4\alpha \kappa \right)}<\gamma <-\frac{2\left(\Delta^{2}-2\alpha \kappa +4\alpha^{2}\kappa \right)}{\alpha \left(-\Delta^{2}+4\alpha \kappa \right)}$, then $c_{r}<A<C_{1}<B_{1}<B_{2}<C_{2}<B_{3}$.

When $-\frac{2\left(\Delta^{2}-2\alpha \kappa +4\alpha^{2}\kappa \right)}{\alpha \left(-\Delta^{2}+4\alpha \kappa \right)}<\gamma <1$, then $c_{r}<C_{1}<A<B_{1}<B_{2}<C_{2}<B_{3}$.

(i) $0<\gamma <\frac{-4\Delta^{2}-3\alpha \Delta^{2}+4\alpha \kappa -12\alpha^{2}\kappa }{\alpha \left(\Delta^{2}+4\kappa +4\alpha \kappa \right)}$

When $c_{r}<c_{n}\leq A$, $\Pi_{S}^{SA}>\Pi_{S}^{SB-1}$.

When $C_{1}\leq c_{n}\leq B_{2}$, let $\Pi_{S}^{SB-2}=\Pi_{S}^{SC}$, $c_{n}=K_{1}=\frac{Q\alpha \left(\Delta^{2}+4\kappa -4\alpha \kappa \right)+\left(-\Delta^{2}+4\left(1+\alpha \right)\kappa \right)c_{r}-\sqrt{\left(\Delta^{2}+4\left(-1+\alpha \right)\kappa \right)\left(\Delta^{2}-4\left(1+3\alpha \right)\kappa \right){\left(-Q\alpha +c_{r}\right)}^{2}}}{8\alpha \kappa }$. When $C_{1}\leq c_{n}<K_{1}$, $\Pi_{S}^{SB-2}>\Pi_{S}^{SC}$; $K_{1}\leq c_{n}\leq B_{2}$, $\Pi_{S}^{SC}>\Pi_{S}^{SB-2}$.

When $B_{2}<c_{n}\leq C_{2}$, $\Pi_{S}^{SC}>\Pi_{S}^{SB-3}$.

When $C_{2}<c_{n}\leq B_{3}$, $\Pi_{S}^{SB-3}<0$.

(ii) $\frac{-4\Delta^{2}-3\alpha \Delta^{2}+4\alpha \kappa -12\alpha^{2}\kappa }{\alpha \left(\Delta^{2}+4\kappa +4\alpha \kappa \right)}<\gamma <-\frac{2\left(\Delta^{2}-2\alpha \kappa +4\alpha^{2}\kappa \right)}{\alpha \left(-\Delta^{2}+4\alpha \kappa \right)}$

When $c_{r}<c_{n}\leq A$, $\Pi_{S}^{SA}>\Pi_{S}^{SB-1}$.

When $C_{1}\leq c_{n}<B_{1}$, $\Pi_{S}^{SB-1}>\Pi_{S}^{SC}$.

When $B_{1}\leq c_{n}\leq B_{2}$, if $\Pi_{S}^{SB-2}=\Pi_{S}^{SC}$, $c_{n}=K_{1}$. When $B_{1}\leq c_{n}<K_{1}$, $\Pi_{S}^{SB-2}>\Pi_{S}^{SC}$; $K_{1}\leq c_{n}\leq B_{2}$,$\Pi_{S}^{SC}>\Pi_{S}^{SB-2}$.

When $B_{2}<c_{n}\leq C_{2}$, $\Pi_{S}^{SC}>\Pi_{S}^{SB-3}$.

When $C_{2}<c_{n}\leq B_{3}$, $\Pi_{S}^{SB-3}<0$.

(iii) $-\frac{2\left(\Delta^{2}-2\alpha \kappa +4\alpha^{2}\kappa \right)}{\alpha \left(-\Delta^{2}+4\alpha \kappa \right)}<\gamma <1$

When $c_{r}<c_{n}<C_{1}$, $\Pi_{S}^{SA}>\Pi_{S}^{SB-1}$.

When $C_{1}\leq c_{n}\leq A$, $\Pi_{S}^{SA}>\Pi_{S}^{SC}$.

When $A<c_{n}<B_{1}$, if $-\frac{2\left(\Delta^{2}-2\alpha \kappa +4\alpha^{2}\kappa \right)}{\alpha \left(-\Delta^{2}+4\alpha \kappa \right)}<\gamma <\frac{-\alpha \left(\Delta^{2}+4\kappa \right)+\sqrt{\alpha^{2}\left(\Delta^{2}+4\left(-1+\alpha \right)\kappa \right)\left(\Delta^{2}-4\left(\kappa +3\alpha \kappa \right)\right)}}{4\alpha^{2}\kappa }$, $\Pi_{S}^{SB-1}>\Pi_{S}^{SC}$. If $\frac{-\alpha \left(\Delta^{2}+4\kappa \right)+\sqrt{\alpha^{2}\left(\Delta^{2}+4\left(-1+\alpha \right)\kappa \right)\left(\Delta^{2}-4\left(\kappa +3\alpha \kappa \right)\right)}}{4\alpha^{2}\kappa }\leq \gamma <1$, if $\Pi_{S}^{SB-1}=\Pi_{S}^{SC}$. When $A<c_{n}<K_{2}$, $\Pi_{S}^{SB-1}>\Pi_{S}^{SC}$; $K_{2}\leq c_{n}<B_{1}$, $\Pi_{S}^{SC}>\Pi_{S}^{SB-1}$.

When $B_{1}\leq c_{n}\leq B_{2}$, if $\frac{-\alpha \left(\Delta^{2}+4\kappa \right)+\sqrt{\alpha^{2}\left(\Delta^{2}+4\left(-1+\alpha \right)\kappa \right)\left(\Delta^{2}-4\left(\kappa +3\alpha \kappa \right)\right)}}{4\alpha^{2}\kappa }\leq \gamma <1$, $\Pi_{S}^{SC}>\Pi_{S}^{SB-2}$. if $-\frac{2\left(\Delta^{2}-2\alpha \kappa +4\alpha^{2}\kappa \right)}{\alpha \left(-\Delta^{2}+4\alpha \kappa \right)}<\gamma <\frac{-\alpha \left(\Delta^{2}+4\kappa \right)+\sqrt{\alpha^{2}\left(\Delta^{2}+4\left(-1+\alpha \right)\kappa \right)\left(\Delta^{2}-4\left(\kappa +3\alpha \kappa \right)\right)}}{4\alpha^{2}\kappa }$, if $\Pi_{S}^{SB-2}=\Pi_{S}^{SC}$, $c_{n}=K_{1}$. When $B_{1}\leq c_{n}<K_{1}$, $\Pi_{S}^{SB-2}>\Pi_{S}^{SC}$; $K_{1}\leq c_{n}\leq B_{2}$, $\Pi_{S}^{SC}>\Pi_{S}^{SB-2}$.

When $B_{2}<c_{n}\leq C_{2}$, $\Pi_{S}^{SC}>\Pi_{S}^{SB-3}$.

When $C_{2}<c_{n}\leq B_{3}$, $\Pi_{S}^{SB-3}<0$.

### Appendix A.3

$\frac{\partial \omega^{DA}}{\partial c_{n}}<0$. $\frac{\partial \omega^{DB-1}}{\partial c_{n}}<0$. $\frac{\partial \omega^{DB-2}}{\partial c_{n}}>0$. $\frac{\partial \omega^{DC}}{\partial c_{n}}<0$.

$\frac{\partial \omega^{DA}}{\partial c_{r}}<0$. $\frac{\partial \omega^{DB-1}}{\partial c_{r}}>0$. $\frac{\partial \omega^{DB-2}}{\partial c_{r}}<0$. $\frac{\partial \omega^{DC}}{\partial c_{r}}<0$.

$\frac{\partial \omega^{DA}}{\partial \Delta }>0$. $\frac{\partial \omega^{DB-2}}{\partial \Delta }<0$. $\frac{\partial \omega^{DC}}{\partial \Delta }>0$.

When $c_{n}<c_{n1}=\frac{-Q\alpha +2c_{r}+Q\alpha \gamma -2Q\alpha^{2}\gamma +2\alpha c_{r}\gamma }{\alpha \left(1+\gamma \right)}$, $\frac{\partial \omega^{DB-1}}{\partial \Delta }>0$. When $c_{n}>c_{n1}$, $\frac{\partial \omega^{DB-1}}{\partial \Delta }<0$.

### Appendix A.4

$\frac{\partial w^{DA}}{\partial c_{n}}>0$. $\frac{\partial w^{DB-1}}{\partial c_{n}}>0$. $\frac{\partial w^{DB-2}}{\partial c_{n}}>0$. $\frac{\partial w^{DC}}{\partial c_{n}}>0$.

$\frac{\partial w^{DA}}{\partial c_{r}}<0$. $\frac{\partial w^{DB-1}}{\partial c_{r}}>0$. $\frac{\partial w^{DB-2}}{\partial c_{r}}>0$. $\frac{\partial w^{DC}}{\partial c_{r}}<0$.

$\frac{\partial w^{DA}}{\partial \Delta }>0$. $\frac{\partial w^{DB-2}}{\partial \Delta }>0$. $\frac{\partial w^{DC}}{\partial \Delta }>0$.

When $c_{n}<c_{n1}$, $\frac{\partial w^{DB-1}}{\partial \Delta }>0$; When $c_{n}>c_{n1}$, $\frac{\partial w^{DB-1}}{\partial \Delta }<0$.

### Appendix A.5

$\frac{\partial q_{n}^{DA}}{\partial c_{n}}<0$. $\frac{\partial q_{n}^{DB-1}}{\partial c_{n}}<0$. $\frac{\partial q_{n}^{DB-2}}{\partial c_{n}}<0$. $\frac{\partial q_{n}^{DC}}{\partial c_{n}}<0$.

$\frac{\partial q_{n}^{DA}}{\partial c_{r}}<0$. $\frac{\partial q_{n}^{DB-1}}{\partial c_{r}}<0$. $\frac{\partial q_{n}^{DB-2}}{\partial c_{r}}>0$. $\frac{\partial q_{n}^{DC}}{\partial c_{r}}<0$.

$\frac{\partial q_{n}^{DA}}{\partial \Delta }>0$. $\frac{\partial q_{n}^{DB-2}}{\partial \Delta }>0$. $\frac{\partial q_{n}^{DC}}{\partial \Delta }>0$.

When $c_{n}<c_{n1}$, $\frac{\partial q_{n}^{DB-1}}{\partial \Delta }>0$; when $c_{n}>c_{n1}$, $\frac{\partial q_{n}^{DB-1}}{\partial \Delta }<0$.

$\frac{\partial q_{n}^{DB-1}}{\partial \Delta }<0$.

$\frac{\partial q_{r}^{DA}}{\partial c_{n}}<0$. $\frac{\partial q_{r}^{DB-1}}{\partial c_{n}}<0$. $\frac{\partial q_{r}^{DB-2}}{\partial c_{n}}>0$. $\frac{\partial q_{r}^{DC}}{\partial c_{n}}<0$.

$\frac{\partial q_{r}^{DA}}{\partial c_{r}}<0$. $\frac{\partial q_{r}^{DB-1}}{\partial c_{r}}<0$. $\frac{\partial q_{r}^{DB-2}}{\partial c_{r}}<0$. $\frac{\partial q_{r}^{DC}}{\partial c_{r}}<0$.

$\frac{\partial q_{r}^{DA}}{\partial \Delta }>0$. $\frac{\partial q_{r}^{DB-2}}{\partial \Delta }<0$. $\frac{\partial q_{r}^{DC}}{\partial \Delta }>0$.

When $c_{n}<c_{n1}$, $\frac{\partial q_{r}^{DB-1}}{\partial \Delta }>0$; when $c_{n}>c_{n1}$, $\frac{\partial q_{r}^{DB-1}}{\partial \Delta }<0$.

### Appendix A.6

$\frac{\partial \Pi_{S}^{SA}}{\partial c_{n}}<0$. $\frac{\partial \Pi_{S}^{SB-1}}{\partial c_{n}}<0$. $\frac{\partial \Pi_{S}^{SB-2}}{\partial c_{n}}<0$. $\frac{\partial \Pi_{S}^{SC}}{\partial c_{n}}<0$.

$\frac{\partial \Pi_{S}^{SA}}{\partial c_{r}}<0$. $\frac{\partial \Pi_{S}^{SB-2}}{\partial c_{r}}>0$. $\frac{\partial \Pi_{S}^{SC}}{\partial c_{r}}<0$.

$\frac{\partial \Pi_{S}^{SA}}{\partial \Delta }>0$. $\frac{\partial \Pi_{S}^{SB-1}}{\partial \Delta }>0$. $\frac{\partial \Pi_{S}^{SB-2}}{\partial \Delta }>0$. $\frac{\partial \Pi_{S}^{SC}}{\partial \Delta }>0$.

When $c_{n}<c_{n1}$, $\frac{\partial \Pi_{S}^{SB-1}}{\partial c_{r}}<0$; when $c_{n}>c_{n1}$, $\frac{\partial \Pi_{S}^{SB-1}}{\partial c_{r}}>0$.

$\frac{\partial \Pi_{M}^{MA}}{\partial c_{n}}<0$. $\frac{\partial \Pi_{M}^{MB-1}}{\partial c_{n}}<0$. $\frac{\partial \Pi_{M}^{MB-2}}{\partial c_{n}}<0$. $\frac{\partial \Pi_{M}^{MC}}{\partial c_{n}}<0$.

$\frac{\partial \Pi_{M}^{MA}}{\partial c_{r}}<0$. $\frac{\partial \Pi_{M}^{MB-1}}{\partial c_{r}}<0$. $\frac{\partial \Pi_{M}^{MB-2}}{\partial c_{r}}<0$. $\frac{\partial \Pi_{M}^{MC}}{\partial c_{r}}<0$.

$\frac{\partial \Pi_{M}^{MA}}{\partial \Delta }>0$. $\frac{\partial \Pi_{M}^{MB-2}}{\partial \Delta }<0$. $\frac{\partial \Pi_{M}^{MC}}{\partial \Delta }>0$.

When $c_{n}<c_{n1}$, $\frac{\partial \Pi_{M}^{MB-1}}{\partial \Delta }>0$; when $c_{n}>c_{n1}$, $\frac{\partial \Pi_{M}^{MB-1}}{\partial \Delta }<0$.

### Appendix A.7

$\frac{\partial q_{n}^{DA}}{\partial \gamma }<0$. $\frac{\partial q_{n}^{DB-1}}{\partial \gamma }<0$. $\frac{\partial q_{r}^{DA}}{\partial \gamma }>0$. $\frac{\partial q_{r}^{DB-1}}{\partial \gamma }>0$. $\frac{\partial \Pi_{M}^{MA}}{\partial \gamma }<0$. $\frac{\partial \Pi_{M}^{MB-1}}{\partial \gamma }<0$.

### Appendix A.8

$\frac{\partial w^{DA}}{\partial \gamma }>0$, $\frac{\partial w^{DB-1}}{\partial \gamma }>0$. $\frac{\partial \omega^{DA}}{\partial \gamma }>0$. $\frac{\partial \Pi_{S}^{SA}}{\partial \gamma }<0$, $\frac{\partial \Pi_{S}^{SB-1}}{\partial \gamma }<0$.

When $\alpha >\frac{1}{2}$, $\frac{-\alpha +\sqrt{8\alpha -7\alpha^{2}}}{2\alpha }<1$. If $\frac{-\alpha +\sqrt{8\alpha -7\alpha^{2}}}{2\alpha }<\gamma <1$, when $c_{n}<c_{n2}=\frac{Q\left(-1+\alpha \right)\Delta^{2}+Q\alpha^{2}\left(-3+2\alpha \right)\kappa +\alpha \kappa \left(Q\alpha \gamma \left(-2+\left(1-2\alpha \right)\gamma \right)+2c_{r}\left(1+\gamma \right)\left(2-\alpha +\alpha \gamma \right)\right)}{\left(-1+\alpha \right)\Delta^{2}+\alpha^{2}\kappa +\alpha^{2}\kappa \gamma \left(2+\gamma \right)}$, $\frac{\partial \omega^{DB-1}}{\partial \gamma }<0$; when $c_{n}\geq c_{n2}$, $\frac{\partial \omega^{DB-1}}{\partial \gamma }>0$.

If $\alpha \leq \frac{1}{2}$, $\frac{-\alpha +\sqrt{8\alpha -7\alpha^{2}}}{2\alpha }\geq 1$. Then $\frac{\partial \omega^{DB-1}}{\partial \gamma }>0$.

### Appendix A.9

The environmental impact is $E=\left(q_{n}\xi_{n}+q_{r}\xi_{r}+\left(q_{n}-q_{r}+q_{r}\right)\xi_{cd}+\left(q_{n}+q_{r}\right)\xi_{u}\right)/\xi_{n}$. After simplifying, the environmental impact is $E=\left(1+y+z\right)q_{n}+\left(x+z\right)q_{r}$. We substitute $q_{n}$ and $q_{r}$ under Decision DA and DB-1 into E, and then obtain $E^{DA}=\frac{\kappa \left(1+y+z+\left(x+z\right)\gamma \right)\left(H_{1}+H_{2}\gamma \right)}{4\kappa +8\alpha \kappa \gamma -\left(\Delta^{2}-4\alpha \kappa \right)\gamma^{2}}$ and $E^{DB-1}=\frac{\left(1+y+z+\left(x+z\right)\gamma \right)\left(\Delta^{2}H_{1}+2\alpha \kappa H_{2}\left(1+\gamma \right)\right)}{4\left(\Delta^{2}+\alpha^{2}\kappa +\alpha \left(\Delta^{2}+2\alpha \kappa \right)\gamma +\alpha^{2}\kappa \gamma^{2}\right)}$.

(i) when $\begin{array}{l} z\geq z_{1}=\frac{H_{2}\left(4\left(1+y\right)\kappa +8x\kappa \gamma +\left(\left(1+y\right)\Delta^{2}-4\left(1-2x+y\right)\alpha \kappa \right)\gamma^{2}\right)}{-H_{2}\left(4\kappa +8\kappa \gamma +\left(\Delta^{2}+4\alpha \kappa \right)\gamma^{2}\right)+H_{1}\left(4\left(-1+2\alpha \right)\kappa -\left(\Delta^{2}-4\alpha \kappa \right)\gamma \left(2+\gamma \right)\right)}\\ +\frac{H_{1}\left(4\left(x-2\left(1+y\right)\alpha \right)\kappa +\left(\Delta^{2}-4\alpha \kappa \right)\gamma \left(2+2y+x\gamma \right)\right)}{-H_{2}\left(4\kappa +8\kappa \gamma +\left(\Delta^{2}+4\alpha \kappa \right)\gamma^{2}\right)+H_{1}\left(4\left(-1+2\alpha \right)\kappa -\left(\Delta^{2}-4\alpha \kappa \right)\gamma \left(2+\gamma \right)\right)} \end{array}$, $\frac{\partial E^{DA}}{\partial \gamma }>0$. When $z<z_{1}$, $\frac{\partial E^{DA}}{\partial \gamma }<0$.

(ii) when, $\frac{\partial E^{DB-1}}{\partial \gamma }>0$. When $z<z_{2}$, $\frac{\partial E^{DB-1}}{\partial \gamma }<0$.
